# Prophage landscapes in clinical MRSA: safety profiling and discovery of Lys81, a broad-spectrum bacteriolytic enzyme

**DOI:** 10.3389/fmicb.2026.1777609

**Published:** 2026-08-31

**Authors:** Fangfei Ran, Minglei Yang, Xiuxiu Zeng, Hui Su, Jiayi Dong, Guosheng Gao, Airong Hu, Yaoren Hu, Yuechao Sun

**Affiliations:** 1 Ningbo No. 2 Hospital, Wenzhou Medical University, Ningbo, Zhejiang, China; 2 College of Veterinary Medicine, Institute of Traditional Chinese Veterinary Medicine, Nanjing Agricultural University, Nanjing, China; 3 MOE Joint International Research Laboratory of Animal Health and Food Safety, College of Veterinary Medicine, Nanjing Agricultural University, Nanjing, China; 4 Department of Thoracic Surgery, Ningbo No. 2 Hospital, Ningbo, Zhejiang, China; 5 Shandong New-Line Biotech Co., Ltd., Weifang, Shandong, China

**Keywords:** collinearity analysis, Lys81, bacteriolytic enzyme, methicillin-resistant *Staphylococcus aureus*, prophage

## Abstract

**Introduction:**

Methicillin-resistant *Staphylococcus aureus* (MRSA) poses a significant threat to global healthcare, requiring novel therapeutic strategies. Prophages, latent phage genomes integrated into bacterial chromosomes, are important resources for antimicrobial development due to their genomic stability and genetic engineering potential.

**Methods:**

In this study, we performed genomewide sequencing on 329 MRSA isolates to predict prophage sequences, followed by analyses of these prophages—including examinations of virulence genes, antibiotic resistance genes, homologous proteins of pathogenic MRSA phages, and functional predictions of these homologous proteins—to evaluate their safety and value as genetic engineering scaffolds and to screen for novel broadspectrum bacteriolytic enzymes.

**Results:**

Our data indicate that 85.7% (282/329) of strains carried complete prophage sequences; 64 strains lacked virulence factors or genes, meeting the core criteria for safe vectors. Resistance screening found only 6 prophages carried *msrA*, confirming the biosafety of the remaining strains. A significant correlation existed between prophage virulence gene capacity and genomic structure (R^2^ = 0.99986684, *p* = 3.64e-69). High-virulence clusters (>10 factors) showed high structural similarity; 10 characteristic sequences linked to *S. aureus* phages and their prevalence patterns were identified via conserved motif analysis. Collinearity analysis with reference to virulent MRSA phages and 3D structural predictions of orthologous proteins identified two lysozymes and a host-recognition device. Notably, Lys81, an N-acetylmuramoyl-L-alanine amidase ortholog, was prioritized and characterized as a broad-spectrum lytic enzyme. Our data show Lys81 has key properties: (1) Broad-spectrum antibacterial activity, lysing 52.3% (23/44) of clinical *S. aureus* strains and cross-acting against Gram-positive bacteria such as *Pseudomonas aeruginosa* and *Listeria*; (2) Excellent environmental adaptability, maintaining activity at pH 5.0 and 0°C, with 25 mM Na^+^ and Ca^2 +^ enhancing function; (3) Potent biofilm clearance, achieving 83% MRSA biofilm reduction at 50 μg/mL; and (4) Favorable in vivo safety/efficacy, eradicating MRSA infections in lung organoid models with minimal cytotoxicity.

**Discussion:**

This study establishes a theoretical foundation for the clinical translation of MRSA prophages, positioning Lys81 as a novel candidate for treating drug-resistant bacterial infections.

## Introduction

Antibiotics, widely utilized for treating bacterial infections, constitute the most recognized and essential class of antimicrobial agents (World Health Organization, 2016; Shoaib et al., 2024). However, antimicrobial resistance is regarded as a major global One Health threat (OECD, 2023). Methicillin-resistant *Staphylococcus aureus* (MRSA), a typical multidrug-resistant pathogen, has developed resistance to multiple classes of antibiotics, leading to limited therapeutic options and increased mortality (O’Neill, 2016; Shoaib et al., 2022). MRSA is classified as a high-priority pathogen by the World Health Organization and remains one of the leading causes of both healthcare-associated and community-acquired infections worldwide (World Health Organization, 2024). The substantial disease burden, high mortality rates, and significant medical costs associated with MRSA underscore the urgent need for alternative and effective therapeutic strategies (Shoaib et al., 2023; Gajdács, 2019). The growing prevalence of antibiotic-resistant bacteria has heightened the urgency to develop innovative therapeutic interventions.

Bacteriophages (phages), which act as natural bacterial killers while exhibiting minimal cytotoxicity toward eukaryotic cells, have emerged as a leading and extensively researched alternative to antibiotic-based treatments (Adamczyk-Popławska et al., 2023). Phages are categorized into two major groups: virulent phages and temperate phages, based on their mode of reproduction within bacterial hosts. Virulent phages exclusively produce progeny through the lytic cycle, whereas temperate phages integrate genomes into the host’s genome and enter a latent state. The latent state of temperate phages integrated into the bacterial genome is referred to as prophages (Hargreaves et al., 2020; Clokie et al., 2011).

Despite prophages cannot directly lyse bacteria like virulent phages, they exhibit clinical potential by being activated under specific signals to regulate bacterial lysis, enabling drug-resistant pathogen clearance with minimal side effects (Bruce et al., 2021; Guo et al., 2024). Notably, prophages represent ideal genetic engineering chassis owing to their inherent genomic stability and amenability to precise manipulation (Clokie et al., 2011). Customized antimicrobial development can be achieved through phage engineering technologies (Pires et al., 2016; Venturini et al., 2022; Zhu et al., 2025). For example, phage Recombineering of Electroporated DNA (BRED) has been used to convert prophages into lytic phages, achieving clinical success in treating drug-resistant *Mycobacterium abscessus* infections (Dedrick et al., 2019).

However, as mobile genetic elements, prophages pose safety risks by transferring virulence factors (VFs) and antibiotic resistance genes (ARGs) via horizontal transfer (Sváb et al., 2022, Peng et al., 2022). Thus, screening prophages free of harmful genes through epidemiological investigations is critical for therapeutic applications (Fortier and Sekulovic, 2013; Jassim and Limoges, 2017; Rahimi and Khashei, 2023).

Against this backdrop, prophage-derived therapies still show significant promise for MRSA treatment: engineered prophages (φSaBov-Cas9-nuc) and prophage-encoded endolysins (LysP108) exhibit potent activity (Park et al., 2017; Lu et al., 2021). Notably, virulent phages such as SLPW also demonstrate significant lytic activity against MRSA *in vitro* (Wang et al., 2016), while animal models validate the *in vivo* safety and efficacy of phage-based interventions (Tuomala et al., 2021). Nevertheless, clinical trials specifically evaluating prophage-based therapies for MRSA remain unreported, highlighting the need for further translational research.

In this study, we collected 329 clinical MRSA isolates and analyzed VFs, AMR genes, and evolutionary relationships in their prophages to evaluate biosafety and functional potential. The aims were to systematically screen safe prophage scaffolds (devoid of AMR/VFs) and identify novel broad-spectrum bacteriolytic enzymes (Lys81, GenBank: PX726137), thereby establishing a foundation for clinical phage therapy development.

## Materials and methods

### Bacterial strains, and growth conditions

All strains utilized in this study are documented in Supplementary Table 1 within the Supplementary material ZIP archive. The plasmid was transferred from the laboratory of Zhejiang University. The strains were grown at 37°C in Brain-Heart Infusion Broth (BHI), unless stated otherwise. The *Escherichia coli (E. coli)* Rosetta (DE3) was cultivated on Lysogeny Broth (LB) medium at 37°C with 180 r.p.m. shaking. The following concentrations of antibiotics were used when necessary: for *E. coli*, kanamycin at 50 μg mL^−1^.

### MRSA strains collection and epidemiological data

780 *S. aureus* isolates and information were collected over the past decade, including sample numbers, dates, sources (sampling departments, sampling sites), and Minimal inhibitory concentration (MIC) values [oxacillin (OXA), linezolid (LNZ), and vancomycin (VAN)]. Clinical drug-resistant MRSA isolates were identified through disk diffusion and dilution methods, selecting suitable MRSA strains.

### Genome sequencing, assembly, and prophage identification

The 329 MRSA isolates were sequenced using the Illumina second-generation high-throughput sequencing platform. After removing sequences with low quality, adapter contamination, and insufficient length, we performed de *denovo* assembly on the clean data. The PHASTEST online platform was utilized to extract prophage genomes from each MRSA genome and identify the integrity (Wishart et al., 2023).

### Virulence factor and gene detection

The virulence factors and ARGs genes were identified in the prophages with intact genomes by MMseqs2 (Steinegger and Söding, 2017). The database used in virulence factor identification is VFDB (database of virulence factors) (Liu et al., 2022). The gene database is CARD (comprehensive ARGs database) (McArthur et al., 2013). After identifying virulence factors and ARGs from prophages, the numbers of these genes will be correlated with prophage and MRSA isolates through Excel data processing.

### Prophage genome dimension reduction and cluster

According to the kmer (*k* = 8) components, prophages were reduced by UMAP (Healy and McInnes, 2024) and clustered by DBSCAN (McArthur et al., 2013). To calculate the *R*^2^, each point was assigned to its nearest core point within the cluster, then the residual sum of squares (RSS) was calculated as the squared distances to the centers. We compared it to the total variance (TSS) and computed *R*^2^ as 1 – RSS / TSS. Prophages carrying 10 or more virulence factors were categorized as the “High-VFs” group, while other prophages were categorized as low-VFs prophages. We assigned high VFs a value of 1 and low VFs a value of 0 and performed Kruskal-Wallis tests on all clustered groups to calculate the *p*-value based on the values. The reference MRSA bacteria were from RefSeq, and the prophages from MRSA were identified by the PHASTEST online platform (Wishart et al., 2023; Agapito et al., 2022).

### Conserved sequence identification in high VFs cluster

Clusters of prophages with an average of more than 10 virulence factors were defined as the high VFs group. Aligning representative motifs of high VFs clusters used the MEME online platform (Bailey and Elkan, 1994)^1^ and used discriminative mode to select 10 motifs from high VFs clusters with low VFs clusters as a control group. Other settings remained default. The conserved motifs of the high VFs clusters were visualized using WebLogo3 (Crooks et al., 2004). The similar sequences of motifs were found, and the organisms and functions of similar sequences were identified by BLAST on NCBI,^2^ PDB,^3^ and UniProt^4^ online platforms with the default setting.

### Selection of MRSA phages for comparative analysis and their lifestyle

Using “MRSA phage complete” as the keywords, we searched the NCBI database^5^ to retrieve all DNA viral sequences. A total of 24 MRSA phage genomes were obtained for comparative analysis with prophages after removal of duplicates. The lifestyle of these phages was predicted by Graphage on the online platform PhageScope (Wang et al., 2024).

### Comparative analysis of representative prophages with MRSA phages

The prophages were categorized into 96 groups by CD-HIT (Fu et al., 2012). Subsequently, representative isolates from each group were selected using CD-HIT. The 96 prophages were then compared and analyzed against the 24 MRSA phages obtained from NCBI earlier. The phylogenetic trees were constructed by the VICTOR web service,^6^ a method for the genome-based phylogeny and classification of prokaryotic viruses (Meier-Kolthoff and Göker, 2017). All pairwise comparisons of the nucleotide sequences were conducted using the Genome-BLAST Distance Phylogeny (GBDP) method (Meier-Kolthoff et al., 2013) under settings recommended for prokaryotic viruses. The resulting intergenomic distances were used to infer a balanced minimum evolution tree with branch support via FASTME, including SPR postprocessing (Lefort et al., 2015), and the calculated formula sum of all identities found in high-scoring segment pairs divided by total genome length. Branch support was inferred from 100 pseudo-bootstrap replicates each. Trees were rooted at the midpoint (Farris, 1974). Due to the limitation of 100 genomes for tree construction, the prophages were divided into two groups equally. Each group was separately subjected to phylogenetic tree analysis in comparison with the 24 MRSA phages. The visualization of the phylogenetic tree was carried out using ITOL.^7^ Based on the phylogenetic trees, six groups of prophages related to the MRSA phages were selected. Each group was individually subjected to phylogenetic tree analysis by the VICTOR web service. The genomes of the six groups were subjected to comparative genomics analysis using Mauve (v 2.4.0) (Darling et al., 2004). The “Align with progressive Mauve” option in Mauve was utilized for aligning the genomes with the phage genomes as references.

### Orthologous protein and function annotation

The phage and prophage groups exhibiting robust homology in the Mauve results were chosen for orthologous protein discovery. We used Proteinortho on the Galaxy online platform (Abueg et al., 2024)^8^ to analyze the homologous proteins between the phages and prophages (Klemm et al., 2023). Subsequently, the Vibrant tool (v1.2.1) was utilized for the phage protein annotation analysis to predict the functions of prophage orthologous proteins (Kieft et al., 2020). Due to the presence of numerous hypothetical proteins in the annotation results, the three-dimensional structures of orthologous proteins between virulent phages with prophages were analyzed and predicted (Jumper et al., 2021). The Clustal Omega service (Sievers et al., 2011) from EMBL’s European Bioinformatics Institute^9^ was employed to eliminate redundancy, focusing exclusively on proteins with sequence similarity below 95%. Three-dimensional structure predictions of the full-length proteins were performed using AlphaFold2 (Goulet and Cambillau, 2021) implemented via the Google Colab environment (Mirdita et al., 2022).^10^ To ensure the reliability of the predicted models, the predicted Local Distance Difference Test (pLDDT) score, a key confidence metric provided by AlphaFold2, was evaluated. Regions with a pLDDT score > 70 were designated as high-confidence structural regions. Structural homology searches and domain identification were conducted using the DALI server^11^ against the PDB database. Structural homologs were identified and ranked based on the highest Z-score, both for the full-length proteins and their manually segmented functional domains (Goulet and Cambillau, 2021; Holm, 2020).

### Bioinformatic analysis of candidate lysozyme genes

Multiple sequence alignment was performed using NCBI BLAST based on the amino acid sequences of candidate lysozyme genes. Conserved functional domains were predicted using NCBI CD-Search, and domain structures were visualized using DOG 2.0 software. Using the Expasy online software^12^ to predict and analyze the physicochemical characteristics of the lyase protein and ascertain its stability.

### Protein expression and purification

To construct the pET28(a)-*Lys81* plasmids, design and synthesize specific primers for *Lys81*-F (CAAATGG GTCGCGGATCCGAATTCATGCAAGCAAAATTAACTAAAAA) and *Lys81*-R (GGTGGTGGTGGTGGTGCTCGAGCTAACTGATT TCTCCCCATAAG) based on the *Lys81* genome. The target fragment was amplified by PCR with the corresponding primers and recovered according to the manufacturer’s protocol of the Gel Extraction Kit (Vazyme). Subsequently, the amplified fragment was digested with *EcoRI* and *XhoI* restriction endonucleases and ligated into the pET28(a) vector. The resultant plasmids were introduced into *E. coli* Rosetta (DE3). Subsequently, strains were cultured at 37°C in LB medium supplemented with 50 μg mL^−1^ kanamycin. Once the optical density at 600 nm (OD_600_) reached 0.6–0.8, protein expression was induced by adding 0.1 mM isopropyl β-D-1-thiogalactopyranoside (IPTG) to the cultures, followed by incubation at 12°C for 48 h. The cells were harvested via centrifugation and resuspended in phosphate-buffered saline (PBS) at pH 7.4. Cell disruption was accomplished through sonication at 30% amplitude, utilizing a 5-s pulse-on and 5-s pulse-off cycle for 20 min at 4°C. Subsequently, the mixture was centrifuged at 12,000 g for 30 min at 4°C to obtain the supernatant.

The soluble supernatant was subjected to SDS-PAGE to verify target protein expression and was subsequently purified using Ni-IDA affinity chromatography with BeyoGold™ His-tag Purification Resin (Fast Flow, denaturant-resistant formulation, Cat. P2236). All purification procedures were performed at 4°C, following the manufacturer’s standard protocols. Briefly, the resin was pre-equilibrated twice with five column volumes of lysis buffer containing 10 mM imidazole, as per the instructions. The protein supernatant was then loaded onto the resin and incubated with gentle rotation for 1–2 h to promote optimal binding between the His-tagged protein and nickel ions. After collecting the flow-through, non-specifically bound contaminants were removed through three rounds of washing with a wash buffer supplemented with 50 mM imidazole. The bound recombinant protein was eluted using an elution buffer containing 250 mM imidazole. All collected fractions (flow-through, wash, and eluate) were analyzed by SDS-PAGE. The concentration of the purified protein was determined using the Bradford assay (Bradford, 1976).

### MIC determination of Lys81

Clinical MRSA isolate (M 56) was cultured in LB medium to a concentration of approximately 1 × 10^8^ CFU/mL. Two fold serial dilutions of the Lys81 stock solution (ranging from 128 to 8 μg/mL) were prepared in LB broth. The inoculum density and incubation conditions for the broth microdilution assay adhered to the recommendations set forth by the Clinical and Laboratory Standards Institute (2024) (CLSI, M07-Ed12, 2024). All assays were conducted in 96-well microplates. A bacterial suspension was added to each well to achieve a final concentration of 5 × 10^5^ CFU/mL. Wells containing bacteria without Lys81 served as growth controls, while blank LB medium without bacteria was used as the background control. The plates were incubated overnight at 37°C. The OD_600_ absorbance was measured using a Multiskan FC microplate reader (Thermo Fisher Scientific, Shanghai, China). The MIC was defined as the lowest concentration of Lys81 that completely inhibited bacterial growth, corresponding to OD_600_ values equivalent to those of the sterile LB blank control.

### Bactericidal activity by CFU reduction assay

The bactericidal activity of Lys81 was determined by CFU reduction assays as previously described (Behera et al., 2024). The reference strain *S. aureus* ATCC 33591 (MRSA) was selected for quantitative bactericidal assay, as it exhibited higher susceptibility to lysozyme with obvious reduction of viable CFU counts, which could fully reflect the bactericidal capacity of lysozyme. Briefly, ATCC 33591 (MRSA) was cultured to an OD_600_ of 0.6 and collected by centrifugation at 3,000 × g for 10 min. The bacterial cells were washed three times with PBS, and then their concentration was adjusted to approximately 1 × 10^8^ CFU/mL. Lys81 was added to the bacterial suspension at a final concentration of 50 μg/mL. PBS buffer was used as a control. After treatment at 37°C for 2 h, the mixture was serially diluted and plated on BHI agar in triplicate. The CFU reduction was calculated following incubation at 37°C for 20 h.

### Bacteriolytic activity by turbidity reduction assay

For the *in vitro* bacteriolytic activity assay, the suspension of clinical isolate (M 56) was adjusted to an OD_600_ of approximately 0.6. Stock solutions of Lys81 with serial concentrations of 60, 80, 100, 120, 140, 160, and 180 μg/mL were prepared and then mixed with an equal volume of bacterial suspension to obtain final working concentrations of 30, 40, 50, 60, 70, 80, and 90 μg/mL. A negative control was established, in which an equal volume of 1 × PBS was mixed with the bacterial suspension. All groups were prepared with three biological replicates, incubated at 37°C with shaking for 2 h, after which OD_600_ values were measured to evaluate the bacteriolytic activity of Lys81.

### Lys81 lytic spectrum assay

A total of 62 bacterial strains were employed for the lytic spectrum determination of Lys81, encompassing *S. aureus*, MRSA, *Acinetobacter baumannii*, *Salmonella enterica* subsp. Enterica *serovar Enteritidis*, *Vibrio vulnificus*, *E. coli*, *Listeria monocytogenes*, *Klebsiella pneumoniae*, and *Pseudomonas aeruginosa*. All tested strains were cultured at 37°C to reach the mid-logarithmic growth phase, centrifuged, and the resulting pellets were washed three times with 1 × PBS. The bacterial cells were then resuspended in 1 × PBS, adjusted to an OD_600_ of ∼0.6, and concentrated to 50% of the initial volume. In 96-well microplates, 100 μL of the concentrated bacterial suspension was mixed with 100 μL of Lys81 at a concentration of 100 μg/mL, resulting in a final Lys81 concentration of 50 μg/mL. while the control group was treated with 100 μL of 1 × PBS instead of Lys81. Each group was configured with three parallel replicates and subjected to incubation at 37°C in a multifunctional microplate reader. The absorbance at OD_600_ was measured at 0 h and 2 h post-incubation, and the OD_600_ reduction rate was calculated to characterize the lytic spectrum of Lys81. Relative lytic activity was quantified using a modified turbidimetric assay originally described by Hawiger (1968). In the original protocol, the percentage decrease in OD_600_ was adopted to quantify the activity of lytic enzymes. The formula used in the present study was revised with PBS blank correction, as follows: Relative lytic activity = (OD_0__*h*_–OD_2__*h*_) (Lys81)/(OD_0__*h*_ – OD_2__*h*_) (PBS) × 100%.

### Lys81 pH stability assay

To evaluate the effect of pH on the lytic activity of Lys81, spectrophotometric OD_600_ turbidity reduction measurements were performed with minor modifications to the previously described protocol (Zhou et al., 2015). In brief, a clinical isolate of MRSA was cultured in BHI medium until it reached the logarithmic growth phase. MRSA suspensions were prepared in 1 × PBS buffer, with pH values adjusted to 4, 5, 6, 7, 8, 9, 10, and 11, to which Lys81 was added, achieving a final concentration of 50 μg/mL. All reaction mixtures were thoroughly homogenized and incubated at 37°C for 120 min. The OD_600_ values of each group were measured at the 0 and 2-h time points of incubation. MRSA suspensions mixed with an equal volume of PBS without Lys81 served as blank controls. The lytic activity of Lys81 under varying pH conditions was quantified by comparing the OD_600_ reduction between the Lys81 treatment groups and the blank control group: A greater magnitude of OD reduction relative to the blank control indicates stronger lytic activity.

### Thermal stability determination

The thermal stability of Lys81 was tested using the similar assay as described previously with modifications (Behera et al., 2024; Shih et al., 1995). Briefly, the clinical MRSA isolate was cultured in BHI medium at 37°C until it reached the mid-logarithmic growth phase. Bacterial cells were harvested via centrifugation, washed three times with sterile 1 × PBS, and adjusted to an OD_600_ of approximately 0.6–0.7 for subsequent lysis assays. Lys81 was prepared as a stock solution at a concentration of 100 μg/mL in 1 × PBS, and aliquots were incubated at various temperatures (4, 16, 25, 37, 42, 56, and 70°C) for 30 min. For each reaction, 100 μL of temperature-treated Lys81 was combined with 100 μL of standardized MRSA suspension, resulting in a final Lys81 concentration of 50 μg/mL in the 200 μL reaction mixture. All reactions were incubated at 37°C for 120 min. A negative control was established by mixing 100 μL of standardized MRSA suspension with 100 μL of 1 × PBS without Lys81. OD_600_ values were recorded at time zero (t_0_) and after 120 min (t) of incubation. The absolute reduction in turbidity (ΔOD_600_) was quantified using the following formula:


ΔOD(t)600=OD(t)600-OD(t)0600.

A larger ΔOD_600_ (t) indicates greater relative lytic activity of Lys81 following thermal treatment. The ΔOD_600_ (t) values from the Lys81 treatment groups were compared with those of the negative control to account for any interference from spontaneous bacterial autolysis.

### Effect of divalent cations and NaCl

The impact of divalent cations and NaCl on the lytic activity of Lys81 was determined as described previously with modifications (Behera et al., 2025). Briefly, NaCl, MgCl_2_, and CaCl_2_ were dissolved in 1 × PBS (pH 7.4) to prepare serial concentrations of 0, 50, 100, 200, and 400 mM, which were used to pre-treat lysozyme. A clinical isolate of MRSA was cultured in BHI medium overnight. Bacterial pellets were harvested by centrifugation and then resuspended in 1 × PBS (pH 7.4). Salt-pretreated Lys81 was mixed with an equal volume of the MRSA suspension to achieve a final Lys81 concentration of 50 μg/mL. After incubation at 37°C for 120 min, the OD_600_ of each sample was measured using a microplate reader. The group treated with 0 mM cations served as the blank control. The bacteriostatic rate was calculated to evaluate the regulatory effect of various ions on Lys81 activity using the formula: Bacteriostatic rate = [(Control OD_600_ - Treatment OD_600_)/Control OD_600_] × 100%.

### Biofilm assays

The biofilm eradication activity of Lys81 against clinical MRSA isolates was quantified through CFU enumeration. In brief, MRSA was cultured in BHI broth at 37°C until reaching the exponential phase. A 100 μL aliquot of the bacterial suspension was diluted with 900 μL of fresh BHI and seeded into 24-well polypropylene plates, followed by static incubation at 37°C for 48 h to facilitate the development of mature biofilms. Control wells containing only BHI were prepared simultaneously. For biofilm clearance testing, the spent medium was aspirated, and each well was gently washed three times with 1 × PBS to remove planktonic cells. Each well was treated with 1 mL of Lys81 solution at a final concentration of 50 μg/mL and incubated at 37°C for 5 h. After treatment, the drug solution was discarded, and the wells were washed twice with PBS to eliminate residual enzyme and detached bacterial fragments. One milliliter of sterile PBS was added to each well, and adherent biofilms were fully scraped off using sterile pipette tips. The suspensions were vortexed for 30 s to thoroughly dissociate biofilm clumps before performing a 10-fold serial dilution. The diluted samples were spread onto agar plates and cultured at 37°C for 24 h. Colony counts were recorded, and log reduction values of viable bacteria were calculated to assess the biofilm-eliminating efficacy of Lys81. All assays were conducted with three independent biological replicates.

### The activity of Lys81 on planktonic cells was verified using the LIVE/DEAD bacterial staining kit and confocal laser scanning microscopy (CLSM)

MRSA clinical isolate was cultured in BHI medium to the logarithmic growth phase. An appropriate volume of the bacterial culture was centrifuged at 10,000 × g for 5 min at room temperature. The supernatant was discarded, and the bacterial pellet was washed once with PBS. The bacterial suspension was then adjusted to a concentration of approximately 1 × 10^8^ CFU/mL (OD_600_ ≈ 0.9) using PBS. The bacterial suspension was treated with Lys81 (final concentration of 50 μg/mL) for 2 h. After treatment, staining was performed using the C2030 Bacterial Viability Staining Kit (DMAO/PI, Beyotime, China) in accordance with the manufacturer’s protocol. Briefly, 1 μL of 100 × staining working solution (prepared by mixing 1 μL PI staining solution, 1 μL DMAO, and 8 μL buffer) was added to every 100 μL of the treated bacterial suspension, and the mixture was gently mixed. The bacterial suspension was then incubated at 37°C in the dark for 15 min. For CLSM observation, 10 μL of the stained bacterial suspension was spotted onto a glass slide (FSL051), and a 24 mm square coverslip (FCGF24) was placed on top. Observations were performed using a confocal laser scanning microscope (Leica STELLARIS STED, Wetzlar, Germany) equipped with a 100 × oil immersion objective (100 × /1.30). The excitation (Ex) and emission (Em) wavelengths were set to 503/530 nm for DMAO (green fluorescence) and 535/617 nm for PI (red fluorescence), with an exposure time of 50 ms. Images were captured using the Leica Application Suite X (LAS X, Leica Microsystems, Wetzlar, Germany) software and a compatible digital camera.

### Isolation and culture of pulmonary organoids

Organoid culture was performed as previously described (Polak et al., 2024). The pulmonary cancer organoid samples were obtained from patients at Ningbo Second Hospital (ethical approval number: (L-nbyjy-ky-2023-011-01). Pulmonary and lung cancer tissue samples were digested and embedded in Matrigel (Corning), then cultured in organoid medium (Biogen Technologies, Suzhou, China). Organoids were passaged weekly: Tryple Express (Gibco, 12604013) was used to digest organoids into single cells at 37°C for 30 min, followed by re-embedding in fresh Matrigel. Organoids with a passage number < 30 were used for experiments.

### Resuscitation and antibacterial assay of pulmonary organoids

Pulmonary organoids were retrieved from liquid nitrogen for resuscitation. Resuscitated pulmonary organoids were seeded into Matrigel droplets in 24-well plates and statically cultured at 37°C with 5% CO_2_ for 24–48 h. After stable spheroid formation, organoids were used for subsequent infection and antibacterial experiments.

An MRSA clinical isolate was inoculated in LB broth and cultured at 37°C with shaking at 200 rpm until the logarithmic phase (OD_600_ ≈ 0.6–0.8). Bacteria were harvested by centrifugation, washed with 1 × PBS, and adjusted to a concentration of 1 × 10^5^ CFU/mL. Pulmonary organoid medium was aspirated and replaced with the MRSA suspension for infection. After 6 h of incubation at 37°C to ensure bacterial adhesion and invasion, non-adherent bacteria were removed by washing 2–3 times with PBS.

Fresh medium was then added for overnight culture, followed by treatment with 1 × MIC Lys81 (64 μg/mL) for 24 h. Organoids were imaged using an ICX41 inverted biological microscope (Sunny Group, China). After imaging, culture supernatants were collected for the lactate dehydrogenase (LDH) cytotoxicity assay using the LDH cytotoxicity assay kit with WST-8 (Beyotime, China, C0018s) according to the manufacturer’s instructions. Absorbance was measured at 450 nm using a microplate reader. Organoids were lysed, serially diluted, and plated for colony counting to evaluate bacterial clearance efficiency. Pulmonary organoid area was measured using ImageJ software to assess tissue damage and repair.

### Statistical analysis

An unpaired student’s *t*-test was used for two-group comparisons, while one-way ANOVA with an LSD *post-hoc* test and two-way ANOVA with Dunnett’s *post-hoc* test were used for multiple-group comparisons as appropriate. All experiments were repeated in at least three independent biological replicates (*n* = 3). Statistical significance was set at *p < 0.05, ^**^p < 0.01, ^***^p < 0.001, and ^****^p < 0.0001, and all analyses were performed using GraphPad Prism 8.0. 2.

### Data availability

The nucleotide sequence of prophage_M81_1 (this study) has been assigned GenBank accession number PX726137 (submission batch: BankIt 3033857). This sequence is currently undergoing GenBank curation and will be publicly released post-review, in line with the associated confidentiality request. Data supporting the findings of this work are available within the paper and its Supplementary Information files. Source data are provided in this paper.

## Results

### Broad distribution of intact prophages in clinical MRSA isolates and their cross-departmental therapeutic potential

**FIGURE 1:**
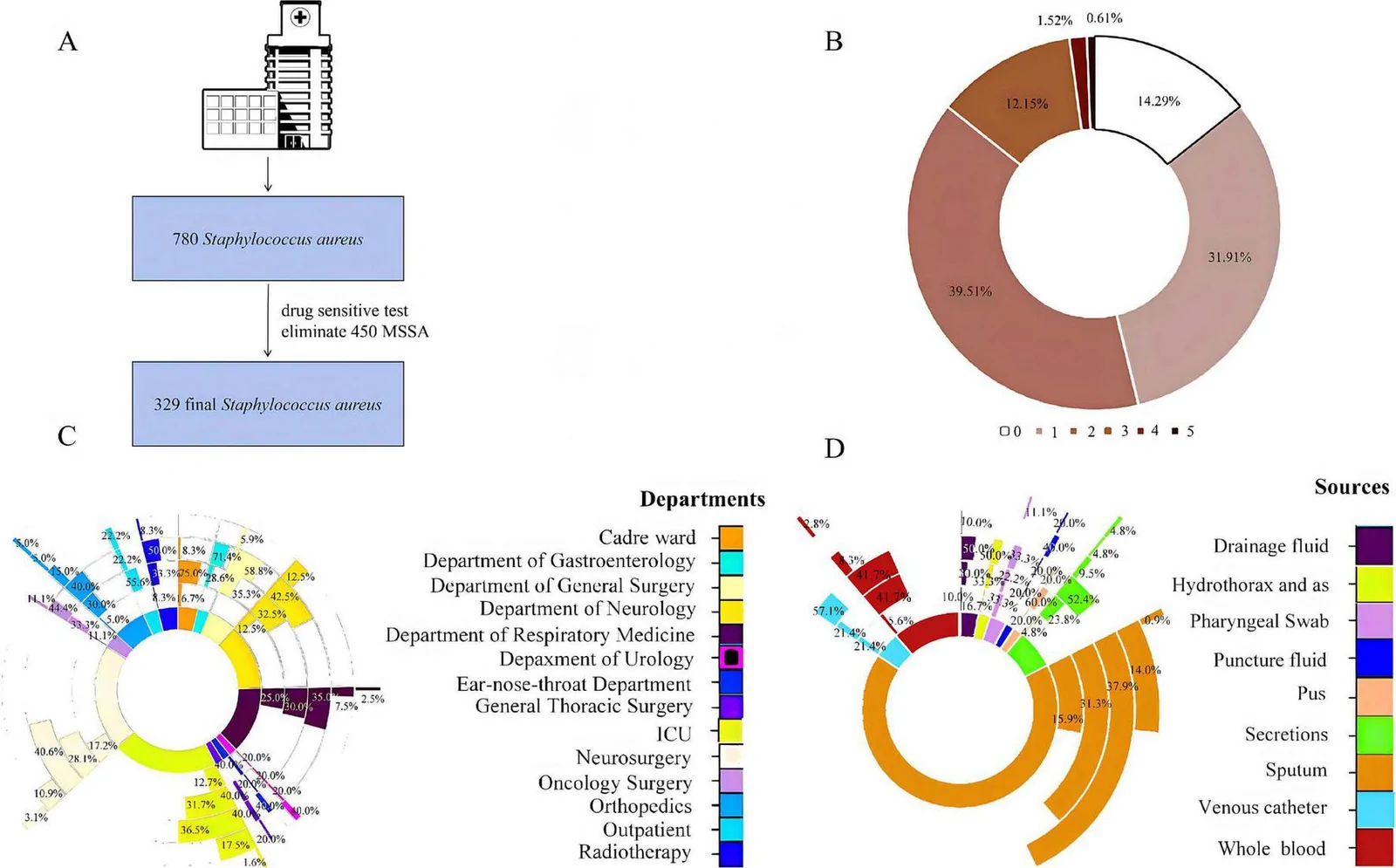
The origin of the MRSA strains and the distribution of complete prophage genomes within the MRSA strains. **(A)** Screening process for MRSA strains. **(B)** The proportion of MRSA strains containing varying numbers of intact prophage genomes. **(C)** The proportion of MRSA strains from different departments containing varying numbers of intact prophage genomes. Rings from the center to the outermost: (1) The proportion of MRSA strains from different departments (2–7) MRSA strains with 0–5 complete prophage regions and rates in the respective departments. **(D)** The number and proportion of MRSA strains from different sources containing varying numbers of intact prophage genomes. Rings from the center to the outermost: (1) The proportion of MRSA strains from different sources (2–7) MRSA strains with 0–5 complete prophage regions and rates in the respective sources. **(C,D)** Due to the limited number of isolates from certain departments and sources, departments and sources with 4 isolates or fewer were excluded from the visualization of prophage profiles.

To evaluate the therapeutic potential of prophages for clinical applications, we systematically collected drug-resistant bacterial strains from clinical settings and characterized the distribution of intact prophage genomes within these isolates. Prophages with intact genomic regions are considered promising candidates for therapeutic development; however, their safety and efficacy require rigorous bioinformatic validation. Here, we collected 780 *S. aureus* strains from hospital settings over a decade, of which 329 were identified as MRSA via antibiotic susceptibility testing (Figure 1A) and subjected to whole-genome sequencing. Intact prophage genomes were identified using the PHASTEST bioinformatic tool for subsequent analysis.

Among the 329 MRSA strains, we detected 436 intact, 79 questionable, and 76 incomplete prophage regions. Reliable prophage regions (intact or questionable) were present in 282 strains (85.7%), with 1–5 regions per strain (median = 2). Notably, 47 strains (14.3%) lacked intact prophages (Figure 1B). Correlation analysis of prophage profiles with clinical departments and sample sources revealed that most MRSA strains across departments and sources harbored 1–2 complete prophage genomes, with > 60% of strains in each category containing intact prophages (Figures 1C,D). These findings indicate that intact prophages are widely distributed in clinical MRSA isolates, supporting their potential applicability for treating MRSA infections across diverse clinical settings.

### Safety assessment of MRSA prophages: virulence factor distribution and impact of virulence gene structure on gene-carrying capacity

**FIGURE 2:**
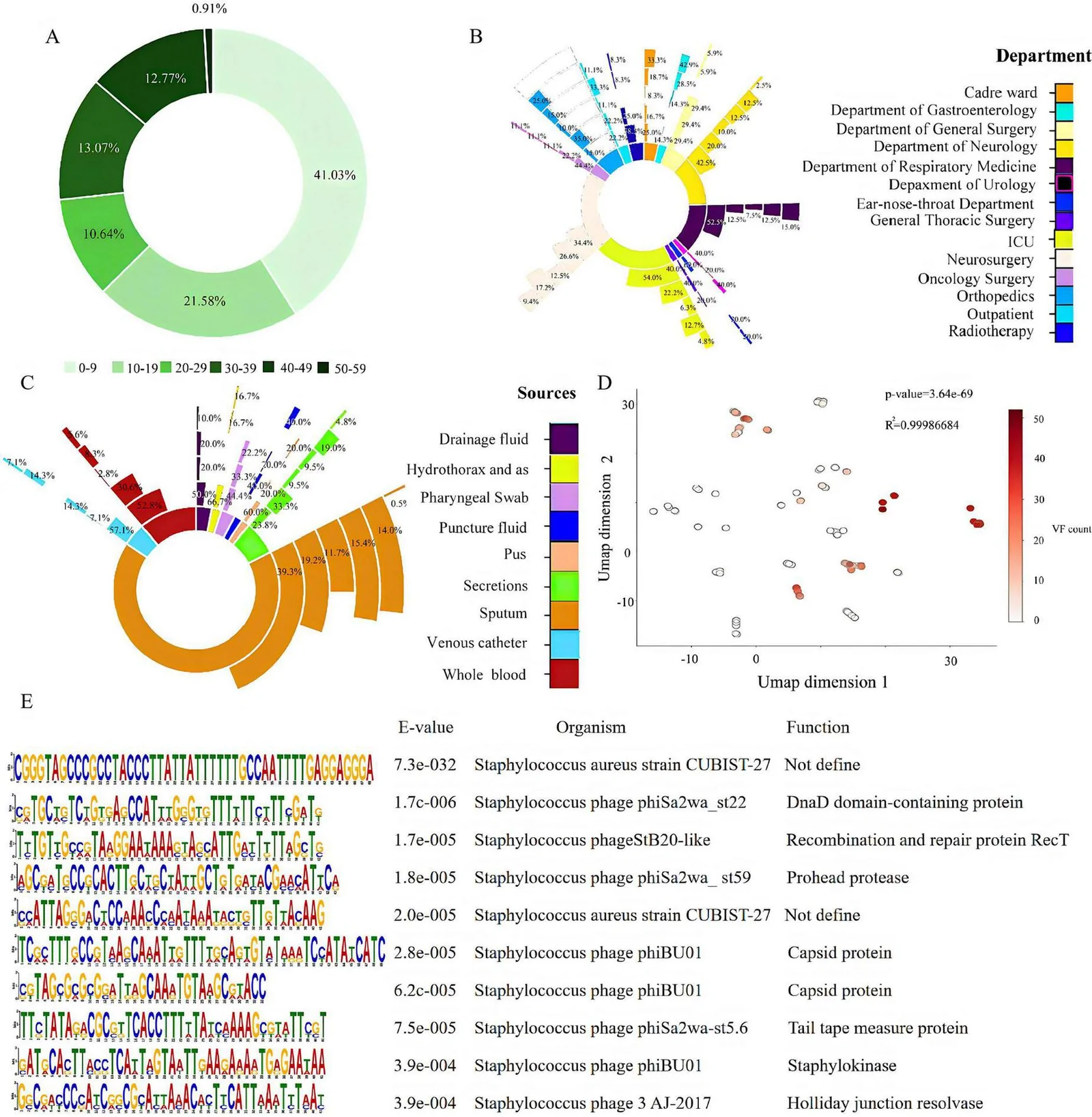
The virulence factors distribution in prophages and the clusters of prophages. **(A)** The proportion of MRSA strains containing varying numbers of virulence factors in prophages. **(B)** The proportion of MRSA strains from different departments containing varying numbers of virulence factors in prophages. Rings from the center to the outermost: (1) The proportion of all MRSA strains from different departments (2–7) MRSA prophage with 0–9, 10–19, 20–29, 30–39, 40–49, 50–59 VFs and rates in the respective departments. **(C)** The number and proportion of MRSA strains from different sources containing varying numbers of virulence factors in prophages. Rings from the center to the outermost: (1) The proportion of all MRSA strains from different sources (2–7) MRSA prophage with 0–9, 10–19, 20–29, 30–39, 40–49, 50–59 VFs and rates in the respective sources. **(B,C)** Due to the limited number of isolates from certain departments and sources, departments and sources with 4 isolates or fewer were excluded from the visualization of prophage profiles. **(D)** The UMAP dimension reduction, clusters of prophages and number of virulence factors in each prophage. Each point in **(D)** is one prophage. Different dot colors reflect the number of virulence factors. The darker the color, the greater the quantity. **(E)** The first 10 consensus motifs of high VFs clusters. The motifs are arranged in descending order from the lowest to the highest based on E-values. The lower the e-value, the higher the confidence level. The height of the letters in **(E)** represents the relative frequency of each base appearing at the specific position, reflecting the conservation of bases at that position. The organisms and functions are predicted by the most similar sequences in databases.

Prophages are capable of increasing the pathogenicity of their host bacteria (Marshall et al., 2021; Andrews et al., 2024; Yates et al., 2024). To verify the safety of prophage therapy, the identification of ARGs and virulence genes is essential. ARGs and virulence genes in the prophages from the isolates were retrieved from the comprehensive CARD and the VFDB. The analysis results reveal a low abundance of ARGs in 515 MRSA prophages, with all six of them listed in Table 1 and 509 prophages containing no AMR. The prophages without AMR cannot transfer AMR from themselves, ensuring that they would not lead to further enhancement of drug resistance in hosts, thereby demonstrating the safety for application. Interestingly, the identified ARGs genes were exclusively *msrA*, suggesting a potential specific pathway for AMR transduction mediated by these prophages in MRSA. 174 prophages contained no VFs, which also demonstrated safety because they were not capable of transferring VFs and improving the virulence of hosts. 5,465 VFs were identified in the prophages (Figure 2A). The result indicated that the sample size with 30–39 VFs and 40–49 VFs in prophages was larger than 20–29 VFs. In other words, the sample quantity did not simply decrease or increase as the number of VFs increased. The same phenomenon was also observed multiple times in isolates from the same department and source, such as cadre ward and sputum (Figures 2B,C).

**TABLE 1:** The summary of prophages of MRSA isolates with antibiotic resistance genes.

Strain	Departments	Sources	Prophage	Antibiotic resistance gene	ARG name
M72	Orthopedics	Secretions	2	1	*msrA*
M141	Orthopedics	Sputum	1	1	*msrA*
M198	Neurosurgery	Sputum	3	1	*msrA*
M268	Neurosurgery	Sputum	4	1	*msrA*
M283	Orthopedics	Secretions	5	1	*msrA*
M358	ICU	Sputum	3	1	*msrA*

According to the genomic structures, the 515 prophage distributions were reduced to two dimensions and clustered (Figure 2D). The figure showed that the prophages with high VFs tended to cluster, indicating the structural similarities between them. The *R*^2^ was 0.99986684, and the *p*-value of VFs between clusters was 3.64e-69, proving the existence of a relationship between VFs counts and genomic structures. The distribution of virulence genes within clustered prophages of similar genomic composition suggested a potential association between the VFs carriage capacity in prophages and their genomic characteristics. The dimension reduction result of 264 prophages within the 118 MRSA strains obtained from NCBI further also supported the conclusion (Supplementary Figure 1).

To identify putative conserved sequences associated with high virulence gene carriage capacity in prophages, we searched for conserved sequences within clusters with high VFs, ensuring that these conserved sequences were not conserved in clusters with low VFs as control groups. The conserved sequences may be related to virulence genes. We identified 10 such motifs and utilized BLAST to determine their biological origins and functions. It was revealed that the organisms of the most similar sequences were *S. aureus* and Staphylococcus phage (Figure 2E). Therefore, the virulence gene carrying capacity is likely associated with the genomic characteristics of *S. aureus* and staphylococcal phages.

### Prophages display collinearity with virulent MRSA phages and share orthologous proteins

**FIGURE 3:**
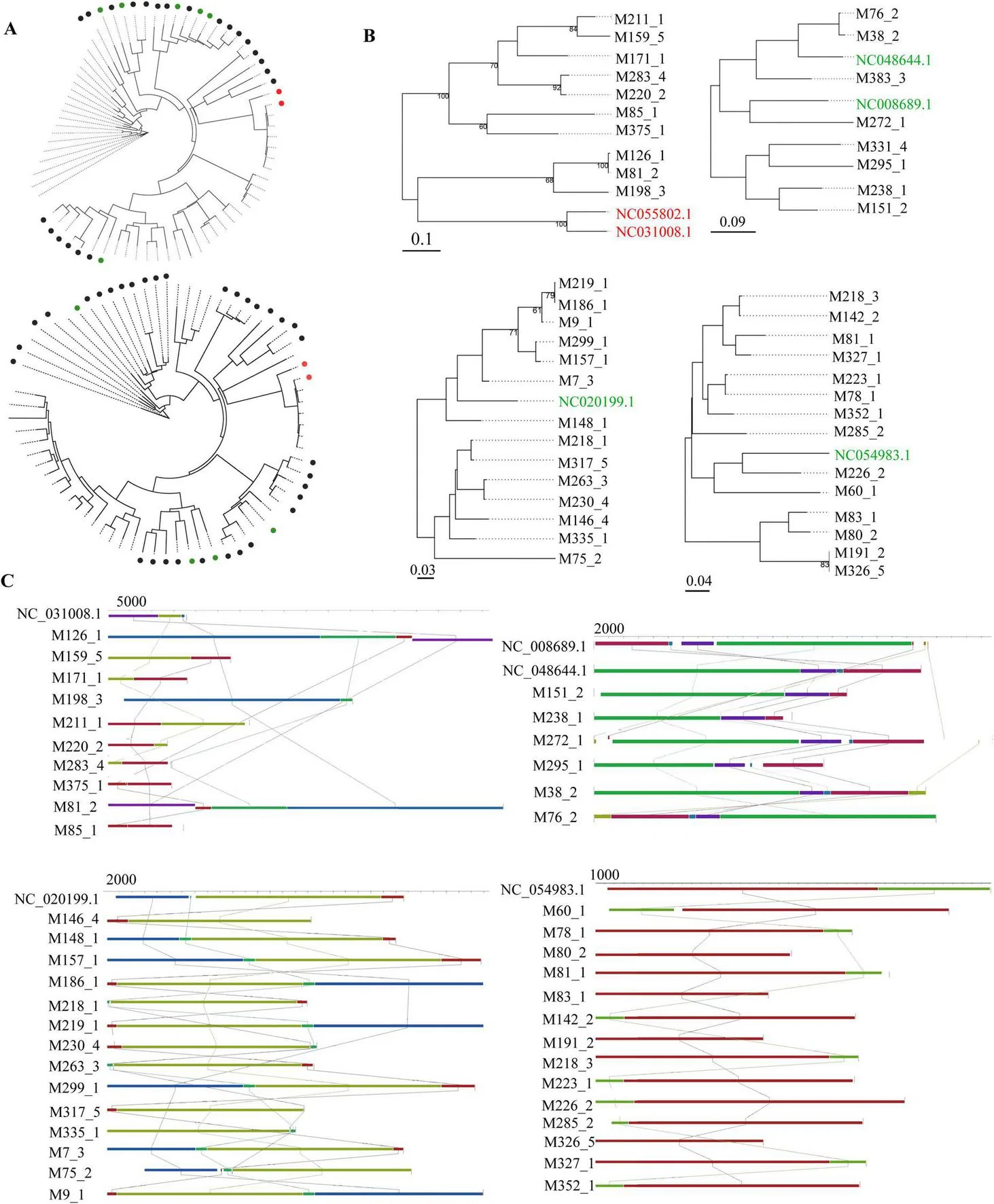
The phylogenetic trees and genome collinearity between prophages with MRSA phages. **(A)** Phylogenetic trees between representative prophages with reference phages. The red dots represent virulent phages, the green dots represent temperate phages, and the black dots represent prophages. **(B)** Phylogenetic trees for reference phages with related prophages used in collinearity analysis. The red labels represent virulent phages, the green labels represent temperate phages, and the black labels represent prophages. **(C)** The collinearity relationship between reference phages with related prophages. In each sub-figure, each group of blocks with the same color represents a set of LCBs. The blocks of the same color in different sub-figures are not related.

Prophage therapy leverages the lysogenization of host bacteria by prophages to guarantee therapeutic efficacy. To predict the infectivity of the prophages, we leveraged the evolutionary relationship between prophages and existing known phages that can infect MRSA. 24 strains of MRSA phages were selected from NCBI, including 8 virulent phages and 16 temperate phages. Owing to the extensive quantity of prophages (515 strains) for analysis, only representative prophages (96 strains) from clusters obtained through CD-HIT clustering were used in phylogenetic analysis. More prophages are closer to temperate phages in evolutionary relationships than virulent phages, and no prophage is on the same branch as virulent phages (Figure 3A). Based on the evolutionary tree result, we selected six groups of related evolutionary phages with MRSA reference phages for comparative analysis.

Evolutionary trees were constructed separately for each group of phages and prophages to display a clear evolutionary relationship. Two prophage isolates appeared on the same branch as the corresponding reference virulent phage, which indicated a close evolutionary relationship between them (Figure 3B). The close evolutionary relationship suggested the potential of prophages for infecting and lysing MRSA as the reference phages.

To investigate the local collinear blocks (LCBs) among each group of genomes, perform gene collinearity analysis separately for the six groups using the MRSA phages as the reference sequences. Among 6 groups, 4 groups have multiple LCBs, indicating the existence of conservative gene blocks (Figure 3C). The analysis result demonstrates that certain prophages exhibit a high degree of homology with the MRSA phage, suggesting the capability to infect MRSA, thereby validating the efficacy of prophage therapy. No corresponding LCB blocks were detected in the other two groups, indicating a lack of homologous genes within these groups. Further analysis will not be conducted with them.

The LCBs indicate the presence of homologous proteins. We utilized Proteinortho to uncover homologous proteins between virulent phages and prophages and annotate the functions. A total of four orthologous proteins were identified, with one annotated as N-acetylmuramoyl-L-alanine amidase (EC 3.5.1.28) (Table 2), an enzyme that facilitates bacterial cell wall degradation (Ghuysen et al., 1969). The presence of homologous proteins between prophages and virulent phages provides molecular-level validation for the effectiveness of prophage therapy.

**TABLE 2:** The orthologous proteins in prophages, corresponding phages and their functions.

Prophage	Position	Reference phage	Predict function
M81_1	17,745–19,643	NC031008.1, NC055802.1, MK936475.1, MK922548.1, MK922547.1, MK922546.1, MK903033.1, MK764384.1	Hypothetical protein
M81_1	27,478–28,923	NC031008.1, NC055802.1, MK936475.1, MK922548.1, MK922547.1, MK922546.1, MK903033.1, MK764384.1	xlyAB; N-acetylmuramoyl-L-alanine amidase [EC:3.5.1.28]
M126_1	2,621–4,123	NC055802.1, MK936475.1, MK922548.1, MK922547.1, MK922546.1, MK903033.1, MK764384.1	Hypothetical protein
M198_3	7,122–8,624	NC055802.1, MK936475.1, MK922548.1, MK922547.1, MK922546.1, MK903033.1, MK764384.1	Hypothetical protein

### Structural prediction and functional annotation of orthologous proteins in MRSA prophages

**FIGURE 4:**
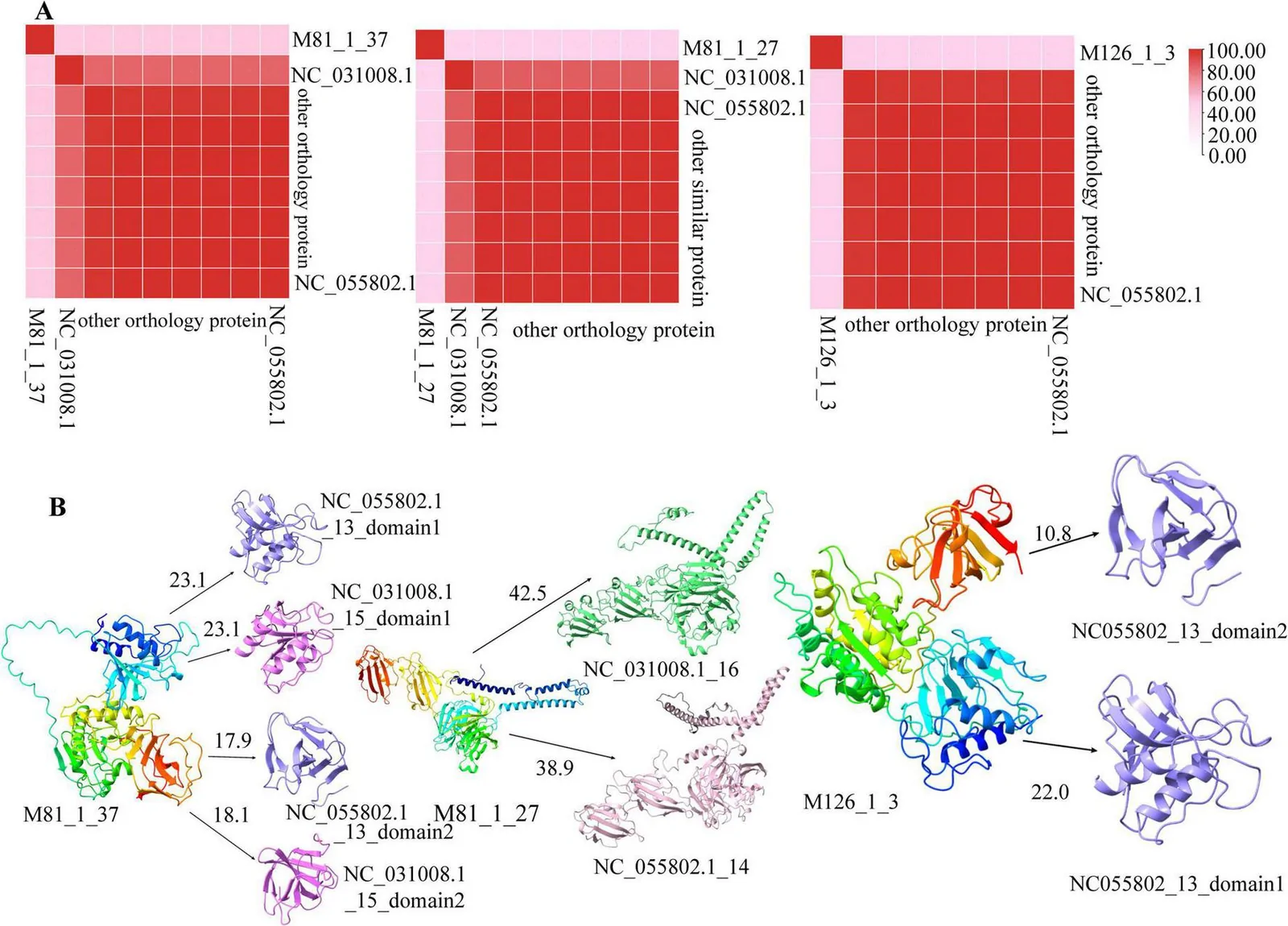
Orthologous protein structure comparison and prediction. **(A)** The heat map shows the similarities between orthologous proteins in prophages and reference phages. The redder cell indicates higher similarity. Proteins with a similarity < 95% will be deduplicated. **(B)** Comparison of predicted protein structures. The tertiary structure of the homologous protein was predicted by AlphaFold2. The N-terminus is represented in blue, while the C-terminus is shown in red. The color gradient visually illustrates the structural folding pathway from the N-terminus to the C-terminus. This model exhibits a tightly folded globular structure, indicating high reliability in structural prediction. The tag M81_1_37 means the 37th protein in the 1st prophage of the M81 sample. The M81_1_37 protein has 3 structural domains: CHAP domain, N-acetylmuramoyl-L-alanine amidase/PGRP domain superfamily, and SH3-like domain. The CHAP domain is like domain 1 of reference phages protein NC_0310081_15 and NC_055802_13, and the SH3-like domain is like domain 2. The number of structural domains between M81_1_27 and the homologous phage proteins is the same. Upon direct comparison, the results indicate a high degree of similarity. The M126_1_3 protein has 3 structural domains: CHAP domain, N-acetylmuramoyl-L-alanine amidase, catalytic domain, and SH3-like domain. The CHAP domain is like domain 1 of reference phage protein NC_055802_13, and the SH3-like domain is like domain 2.

To reduce analytical redundancy, the nucleotide sequences of these four proteins were compared and deduplicated against the homologous protein sequences in the reference phages. The nucleotide sequences corresponding to the orthologous proteins in M126_1_3 and M198_*3* are entirely identical. After deduplicating the remaining sequence comparisons, seven non-redundant results were obtained. The similarity between the sample and reference sequences is illustrated in Figure 4A.

The structures of seven proteins were predicted using AlphaFold2 and subsequently compared. For orthologous protein groups with varying numbers of structural domains, the domains were segmented and analyzed individually. The M81_1_27 (Lys81) protein comprises three structural domains: The CHAP domain, the N-acetylmuramoyl-L-alanine amidase/PGRP domain superfamily, and the SH3-like domain. The CHAP domain exhibits similarity to domain 1 of reference phage proteins NC_0310081_15 and NC_055802_13, while the SH3-like domain corresponds to domain 2. Notably, the number of structural domains in M81_1_27 matches that of its homologous phage proteins, indicating a high degree of similarity upon direct comparison. The M126_1_3 (M126_1) protein also contains three structural domains: the CHAP domain, the N-acetylmuramoyl-L-alanine amidase catalytic domain, and the SH3-like domain. The CHAP domain shows similarity to domain 1 of reference phage protein NC_055802_13, and the SH3-like domain corresponds to domain 2 (Figure 4B). The analysis results indicate that proteins within the prophages exhibit structural similarities to those in the reference phages, thereby demonstrating the conservation of orthologous proteins.

Based on the predicted three-dimensional structures of the proteins, we utilized the Dali server to identify proteins with similar structures for functional prediction. The annotation result for the protein in M81_1 (position: 27,478–28,923) aligns well with the predicted protein, the N-acetylmuramoyl-L-alanine amidase domain (PDB: 4ols-D) (Gu et al., 2014). The hypothetical protein in M81_1 (position: 17,745–19,643) resembles the host-recognition device of *S. aureu*s phage Phi11 (PDB: 5efv-A) (Koc et al., 2016), while the other hypothetical protein in M126_1 (position: 2,621–4,123) has been identified as lysozyme (PDB: 4olk-A) (Gu et al., 2014). The lysozyme may potentially exhibit antibacterial effects against MRSA.

### Sequence homology and functional domain analysis of candidate lysozymes

To validate the annotation results, sequence alignment analyses were conducted using NCBI BLAST. The multiple sequence alignment and homology analysis demonstrated a high sequence identity between M126_1/M81_1 and known staphylococcal lysozymes. Specifically, M126_1 exhibited over 97% sequence identity with homologous sequences (WP_070861812.1, WP_368864113.1; Supplementary Table 2). Lys81 displayed 82–99% homology with reported endolysins (WP_165811754.1, WP_031784479.1, AWD93103.1; Supplementary Table 3) and featured a modular CHAP-Ami_2-SH3_5 domain architecture (Supplementary Figure 2). Both M126_1_3 and M81_1_37 are classified within the phage endolysin family based on their domain architecture. Sequence comparisons revealed low homology (28%) between M126_1_3 and M81_1_37, suggesting their classification as distantly related homologs or functionally divergent proteins with significant divergence in their full-length amino acid sequences (BLASTP E-value = 1.2) (Supplementary Table 4). This divergence primarily arises from subfamily-level sequence drift and hypervariable interdomain linkers, rather than from protein-level non-homology. Phylogenetic analysis positioned M126_1_3 in a basal clade (100% bootstrap support), forming a sister group with the main clade that includes M81_1 and other distant members, thereby identifying M126_1_3 as an early diverging novel subtype (Supplementary Figure 3 and Supplementary Table 4).

Given the recent identification of M126_1_3 and its limited sequence matches in public databases, Lys81 has been prioritized due to its canonical CHAP-Amidase_2-SH3_5 architecture. Lys81 was selected for its complete and high-confidence domain annotation, which is consistent with characterized staphylococcal phage lysozymes (Golban et al., 2025). The well-defined domain boundaries and extensive functional validation further facilitate subsequent expression experiments (Supplementary Figure 2). Physicochemical characterization of the *Lys81* (*M81_1_37*) gene product demonstrated that the encoded protein consists of 482 amino acid residues, with a calculated molecular weight of 54,116.55 Da, a theoretical isoelectric point (pI) of 8.91, a mean hydrophilicity coefficient of -0.475, and an instability index of 37.62 (Table 3). These favorable properties confirm that M81_1 is a stable alkaline protease, making it suitable for functional studies.

**TABLE 3:** Physicochemical properties of lysozyme.

Protein ID	Molecular weight	Isoelectric point	Grand average of hydropathicity	Instability index
M81_1_37	54116.549	8.906645775	-0.474844075	37.61891892

### Production and antibacterial characterization of Lys81

**FIGURE 5:**
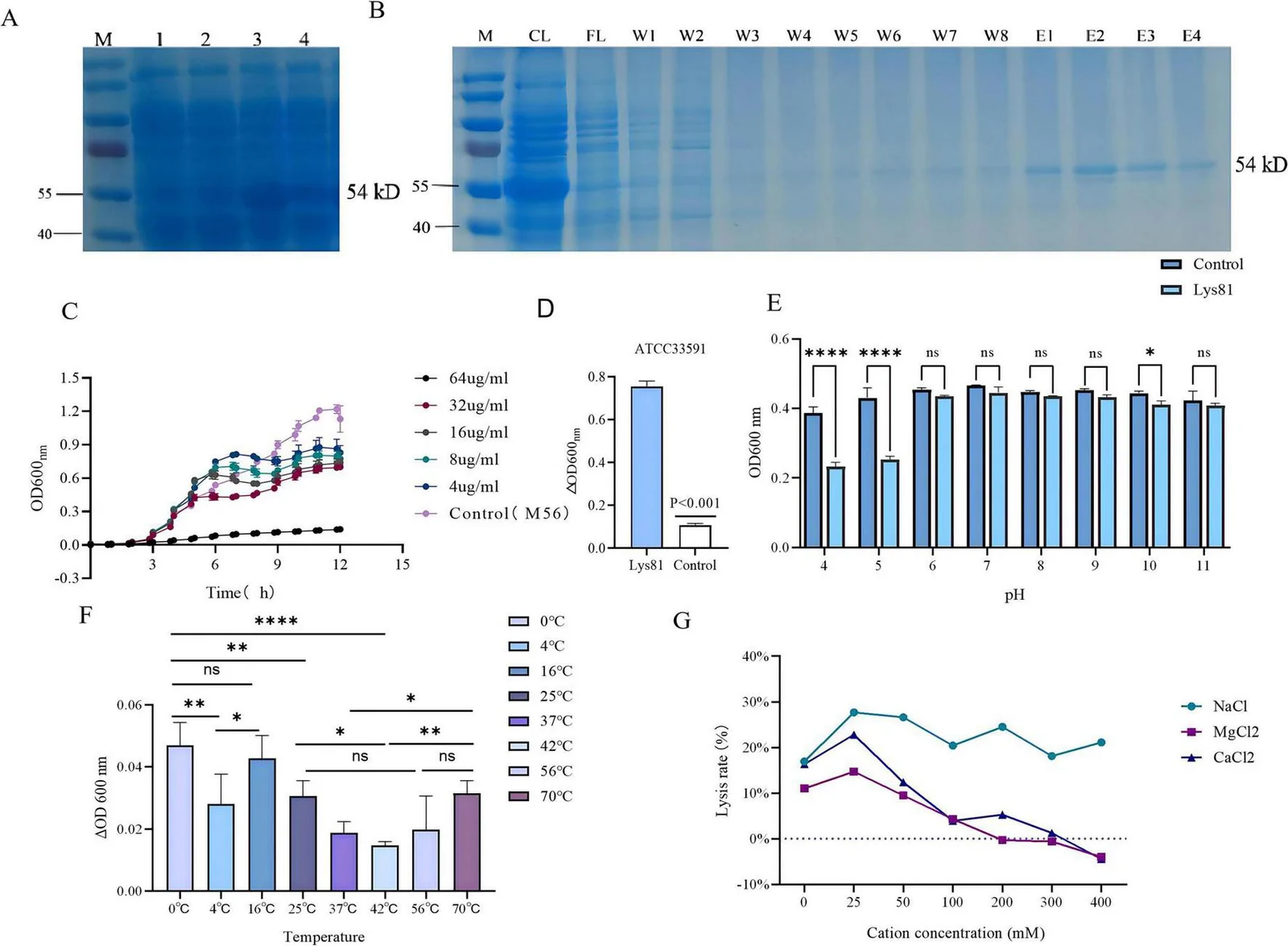
Expression, purification, and *in vitro* bacteriolytic activity analysis of Lys81. **(A)** SDS-PAGE analysis of Lys81 prokaryotic expression. M, Protein marker; Lane 1, Uninduced bacterial lysate; Lane 2, Supernatant of uninduced bacterial lysate; Lane 3, Induced bacterial lysate; Lane 4, Supernatant of induced bacterial lysate. **(B)** M, Protein marker; CL, Bacterial lysate; L, Lysate supernatant; FT, Flow-through; W1–W3, Initial washes; W4–W5, Washes with 10 mM imidazole; W6–W8, Washes with 20 mM imidazole; E1–E4, Elutions with 250 mM imidazole. **(C)** Determination of minimum inhibitory concentration (MIC) of Lys81 against clinical MRSA isolate by growth curve assay. **(D)** Lytic activity of Lys81 against the reference strain expressed as ΔOD_600_. Statistical significance was analyzed by Student’s *t*-test. **(E)** pH-dependent activity of Lys81 following 2-hour incubation with MRSA. Asterisks indicate significant differences between groups (*P < 0.05, ***P < 0.001). *P*-values were calculated by two-way ANOVA with Dunnett’s post hoc test from three independent biological duplicates. **(F)** Temperature stability of Lys81. Asterisks indicate significant differences between groups (*P < 0.05, **P < 0.01, ***P < 0.001, ****P < 0.0001). *P*-values were determined by one-way ANOVA with LSD test, and mean differences (95% confidence intervals) were calculated from three independent biological duplicates. **(G)** Effect of cations on Lys81 activity. Lysis rate was calculated as [(OD_600_ control–OD_600_ treatment)/OD_600_ control] × 100%. Control group: Lys81 without cations; Treatment groups: Lys81 with various concentrations of Na^+^, Mg^2+^, or Ca^2+^.

The selected lysin gene, designated Lys81, encodes a ∼54 kDa protein comprising an N-terminal CHAP catalytic domain, a central Amidase-2 domain, and a C-terminal SH3_5 cell-wall-binding domain (Supplementary Figure 2). To characterize the bacteriolytic activity of Lys81, the target gene was amplified from prophage M81_1, resulting in a 1,492 bp amplicon (Supplementary Figure 4A). This fragment was subsequently cloned into the pET-28(a) vector (Supplementary Figure 4C), and recombinant expression was induced in *E. coli* Rosetta (DE3) using 0.1 mM IPTG at 12°C for 48 h. Affinity purification yielded a protein of approximately 54 kDa, which predominantly accumulated in inclusion bodies, with only minor amounts present in soluble forms (Figures 5a,b).

Given that clinical isolates represent the actual target pathogens for potential clinical applications, stability and activation assays were performed using clinical strains to reflect practical performance, while the reference strain *S. aureus* ATCC 33591 served as a positive control to verify the inherent enzymatic function. The MIC of Lys81 against the target clinical MRSA isolate was determined to be 64 μg/mL using the two-fold broth microdilution method, and this result was further validated by growth curve assays (Figure 5C).

To determine the optimal working concentration, a concentration gradient was established based on MIC values for *in vitro* lytic activity assays. Compared to the control group, bacterial suspensions treated with varying concentrations of Lys81 exhibited a progressive decrease in OD_600_, accompanied by gradual clarification of the culture medium due to bacterial lysis, whereas the control group maintained stable OD_600_ throughout the observation period. Specifically, 30 μg/mL induced only a slight OD_600_ reduction, while 40 and 50 μg/mL treatments showed continuous downward trends. Notably, 50 μg/mL Lys81 achieved maximal lytic efficacy, with activity comparable to 60–180 μg/mL concentrations (Supplementary Figure 5). The lytic activity of 50 μg/mL Lys81 was further validated using a reference strain *S. aureus* ATCC 33591, demonstrating a significant reduction in ΔOD_600_ (Figure 5D). Time-dependent lytic curves of the reference strain *S. aureus* ATCC 33591 further corroborated this dynamic process (Supplementary Figure 7). A similar effect was observed in a clinical isolate (M172), where Lys81 reduced OD_600_ from approximately 0.9 to below 0.2 within 2 h (Supplementary Figure 8). Collectively, these results confirm the robust lytic activity of Lys81.

The lytic spectrum of Lys81 was further evaluated against 62 bacterial strains, revealing broad bacteriolytic activity. Specifically, Lys81 effectively targeted 52.3% of the tested *S. aureus* isolates, as well as *V. vulnificus*, *L. monocytogenes*, and 50% of *P. aeruginosa* isolates. Notably, the *P. aeruginosa* type strain exhibited over 30% bacteriolytic efficiency. In contrast, no activity was detected against *A. baumannii*, *S. Enteritidis*, *E. coli*, or *K. pneumoniae* (Supplementary Table 4).

### pH-dependent activity, thermostability, and Na^+^-mediated activation of Lys81

After determining the optimal concentration, we evaluated the temperature stability (Figure 5E), pH stability (Supplementary Figure 4), and cation activation capability (Figure 5G) of lysozyme in clinical isolate. Lys81 exhibits pH-dependent activity, with peak lytic activity at pH 5.0, followed by a gradual decrease in activity as pH increases beyond this point (Figure 5E). The activity of Lys81 displayed a non-monotonic dependence on the 30-min pre-incubation temperature (Figure 5F). Pre-incubation at 0°C yielded the highest lytic activity, followed by a secondary peak at 16°C. The activity at 4 and 25°C was statistically indistinguishable but significantly lower than that at 16°C (*p* < 0.05). A sharp decline in activity was observed within the physiological range (37 and 42°C), with the lowest lytic efficiencies recorded at these temperatures, showing no significant difference between them. Unexpectedly, pre-incubation at 56 and 70°C restored activity to levels statistically comparable to those at 25°C. This non-monotonic profile suggests that brief thermal priming induces distinct, stable conformational states in the enzyme (Min et al., 2006; English et al., 2006).

Cations are known to differentially modulate lysozyme activity. In this study, the lysis rate of Lys81 was determined to be 16.94% under sodium (Na^+^)-deficient conditions. Notably, supplementation with sodium concentrations ranging from 25 to 400 mM significantly enhanced its lytic activity compared to the Na^+^-free control, thereby confirming a potent activating effect of Na^+^ on Lys81 (Figure 5G). The maximum bacteriolytic activity was observed at a Na^+^ concentration of 25 mM, with the lysis rate reaching 27.69%. However, a gradual decrease in the activating effect was noted with further increases in Na^+^ concentration beyond 50 mM, suggesting a concentration-dependent activation profile of Na^+^. In contrast to Na^+^, 25 mM magnesium (Mg^2 +^) did not significantly affect the lytic rate of Lys81, whereas elevated Mg^2 +^ concentrations markedly reduced lytic activity below that of the Mg^2 +^-free control, indicating strong enzyme inhibition. The activating effect of calcium (Ca^2 +^) on Lys81 mirrors the trend observed with Na^+^. In the absence of supplementary Ca^2 +^, the lysis rate of Lys81 is 16.34%. Compared to the Ca^2 +^-free control group, the lysis rate significantly increases to 22.82% when the Ca^2 +^ concentration is 25 mM, confirming the activating effect of Ca^2 +^ on Lys81. However, as the Ca^2 +^ concentration rises to between 50 and 400 mM, the lysis rate exhibits a significant concentration-dependent decrease, indicating that elevated levels of Ca^2 +^ exert a notable inhibitory effect on the activity of Lys81. These findings suggest that different cations exert differential activating effects on Lys81.

### Strain-dependent bactericidal activity, membrane damage and biofilm eradication of Lys81

**FIGURE 6:**
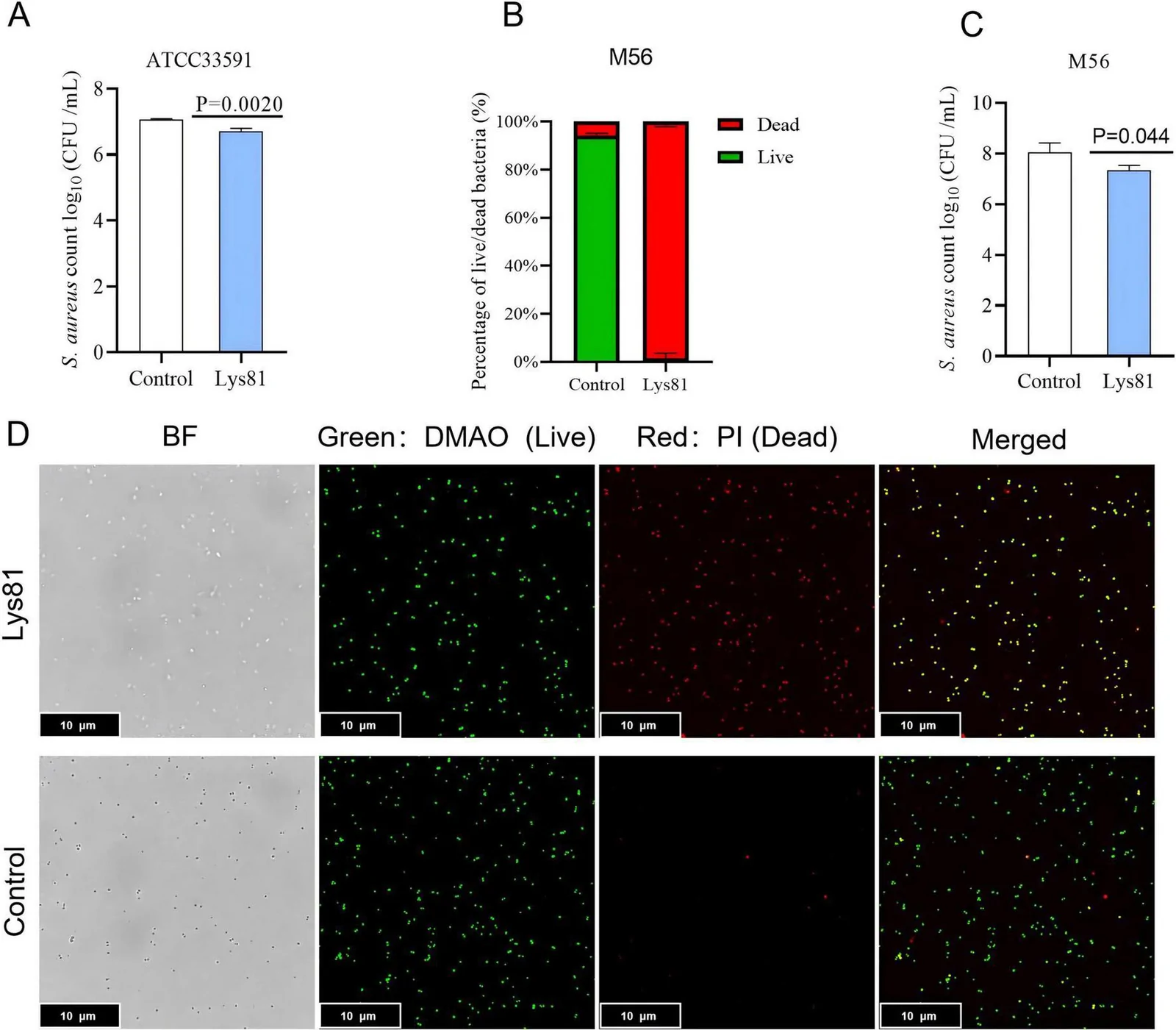
Strain-dependent bactericidal activity, membrane damage, and biofilm eradication capacity of Lys81**. (A)** Bactericidal activity of Lys81 against the reference strain *S. aureus* ATCC 33591 determined by CFU counting, showing a significant reduction in bacterial counts. **(B)** Quantitative analysis of LIVE/DEAD staining for clinical MRSA isolate, showing the proportion of dead bacteria after lysozyme treatment. A significant increase in the dead bacteria ratio was observed. **(C)** Biofilm eradication capacity of lysozyme against clinical MRSA isolate, assessed via biofilm-associated CFU counting. A remarkable reduction in viable bacteria within biofilms was noted. **(D)** Representative LIVE/DEAD fluorescent images of clinica lMRSA isolate after lysozyme treatment. Increased red fluorescence signals indicate bacterial membrane damage and dead bacteria. From left to right: brightfield, green fluorescence, red fluorescence, and merged images. Scale bar: 10 μm. Data are presented as mean ± SD. Statistical significance was analyzed by Student’s *t*-test.

Although Lys81 significantly reduced the OD_600_ and clarified the bacterial solution of both clinical MRSA (M 56) and reference strains, the results of this study demonstrated that its bactericidal activity is strain-dependent. Specifically, after 2 h of Lys81 treatment, the OD_600_ of the reference strain ATCC 33591 decreased by approximately 0.78, and the CFU decreased by 0.35 log10 units. In contrast, while the clinical strain exhibited a significant decrease in OD_600_ (Supplementary Figures 7, 8), there was no statistically significant difference in CFU. To investigate the mechanism underlying the OD_600_ reduction in clinical strains, a LIVE/DEAD staining assay revealed that after 2 h of Lys81 treatment, the mortality rate of clinical strains increased from < 10% in the control group to 98% (Figure 6B), accompanied by evident membrane structural damage and an enhanced red fluorescent signal (Figure 6D). This suggests that Lys81 primarily exerts specific membrane-damaging effects on clinical strains. Further biofilm clearance experiments confirmed that Lys81 effectively eliminates biofilms formed by clinical isolates, resulting in a significant reduction in biofilm-associated CFU, with a decrease of approximately 0.69 log_10_ units in CFU after 5 h of exposure (Figure 6C). In conclusion, Lys81 exhibits strain-dependent antibacterial effects: it demonstrates potent bactericidal activity against reference strain ATCC 33591, primarily induces membrane damage to clinical isolates, and possesses efficient biofilm clearance capabilities.

### Antibacterial efficacy and organoid-type-dependent cytotoxicity of lysozyme in 3D pulmonary organoid models

**FIGURE 7:**
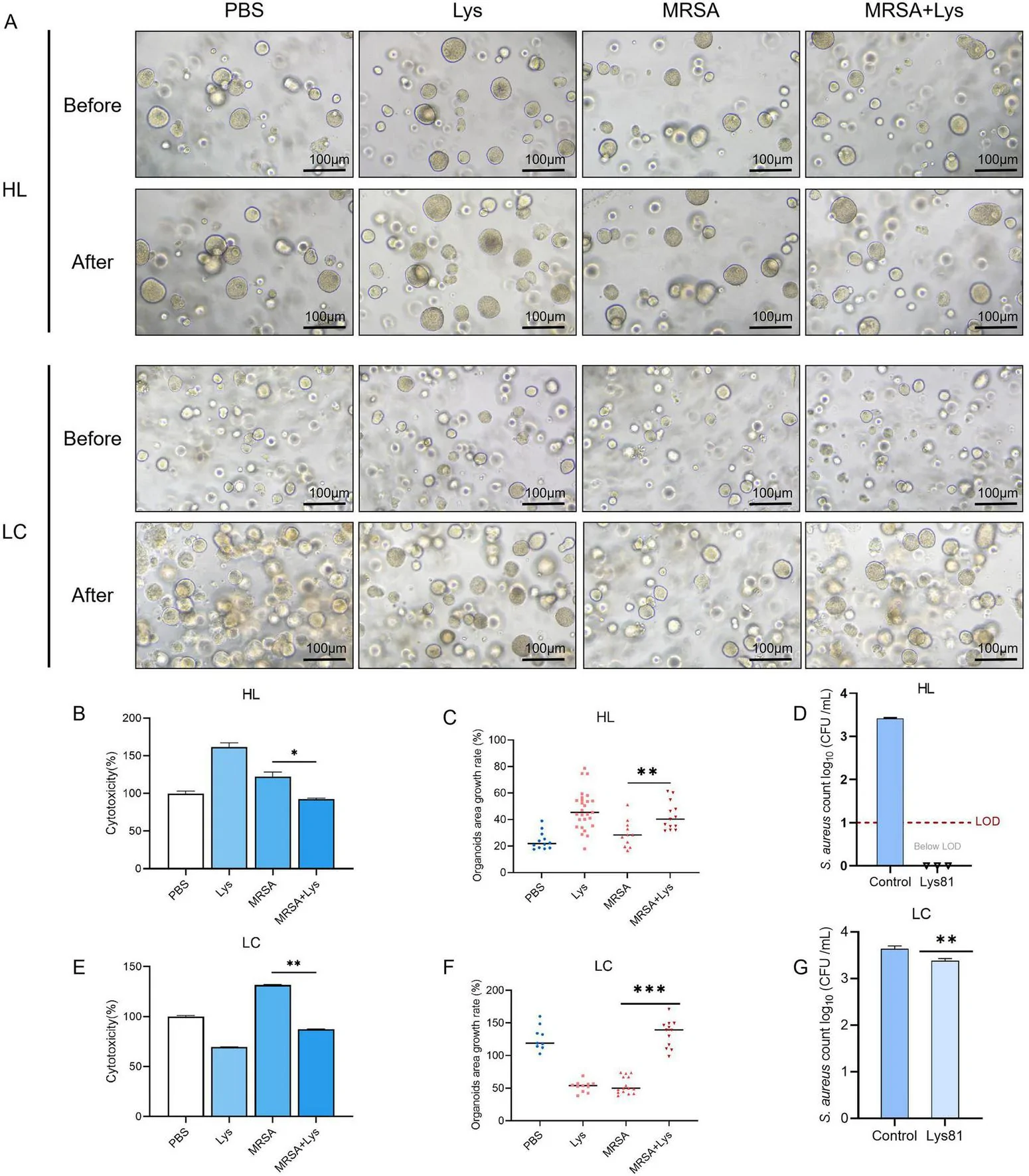
Antibacterial efficacy and organoid-type-dependent cytotoxicity of Lys81 in pulmonary 3D organoid models. Scale bar: 10 μm. HL, normal human pulmonary organoids; LC, lung cancer-derived pulmonary organoids. **(A)** Representative images of normal and cancer-derived pulmonary organoids following Lys81 treatment and MRSA infection. **(B,E)** LDH release assay for evaluating Lys81-mediated cytotoxicity. Lys81 induced mild cytotoxicity in normal pulmonary organoids, while cytotoxicity was attenuated in cancer-derived organoids; reduced LDH leakage was detected in MRSA-infected normal organoids. Data are shown as mean ± SD. **(C,F)** Quantification of organoid area growth rate reflecting tissue injury and repair after Lys81 intervention. Each dot represents an independent biological replicate, and horizontal lines indicate the median value of each group. The number of biological replicates varied across groups due to differences in organoid survival during culture. **(D,G)** Log*10* CFU counts of MRSA recovered from organoids to assess the bactericidal capacity of Lys81. The red dashed line denotes the limit of detection (LOD). Samples with values below LOD were excluded from statistical analysis. Data are presented as mean ± SD. Statistical analysis: One-way ANOVA followed by Dunnett’s *post-hoc* test was used for intergroup comparisons in **(B,C,E,F)**; an unpaired two-tailed Student’s *t*-test was applied to compare two groups in panels D and G. *P < 0.05, **P < 0.01, ***P < 0.001.

To evaluate the *in vivo*-like antibacterial efficacy and biocompatibility of Lys81, we established MRSA-infected pulmonary organoid models derived from both normal and cancerous tissues, subsequently treating them with Lys81 at its MIC (Figure 7). In normal organoids, treatment with Lys81 resulted in a higher release of LDH compared to other groups, indicating mild cytotoxicity. Despite the elevated LDH levels, the area of normal organoids was significantly larger than that of the other groups, suggesting a potential compensatory proliferation or repair response induced by mild cellular injury. Notably, in MRSA-infected normal organoids, Lys81 significantly reduced LDH release, thereby alleviating infection-induced tissue injury. No viable bacteria were detected post-treatment, which may be attributed to the preservation of tissue structure and a favorable microenvironment that enhances the antibacterial activity of Lys81 (Figures 7B–D). In cancer-derived organoids, Lys81 exhibited lower cytotoxicity than the control group. Although Lys81 significantly decreased the bacterial burden in infected cancer organoids, the reduction in CFU was less pronounced than that observed in infected normal organoids (only 0.25 log10 CFU reduction) (Figures 7E–G). These results demonstrate that Lys81 exerts effective antibacterial activity in infected organoids and exhibits organoid-type-dependent cytotoxicity.

## Discussion

The rising prevalence of antibiotic-resistant bacterial infections has spurred a resurgence in bacteriophage therapy research. Furthermore, phage therapy is being investigated as a strategy to modulate commensal microbiota communities through targeted bacterial elimination. In this context, the development of phages and lysozymes has become a key research focus, driven by two critical advantages of lysozymes: their ability to specifically disrupt bacterial cell walls and their low potential for resistance development. Consequently, numerous lysozymes and perforins are currently under investigation for the treatment of drug-resistant bacteria. However, limited studies have focused on the endolysins derived from MRSA prophages (Kim et al., 2020; Khan et al., 2024). In the present study, we analyzed prophages from 329 MRSA clinical isolates, revealing that 85.7% carried intact prophages, with 64 strains lacking virulence factors or resistance genes, making them suitable as genetic engineering scaffolds. Cluster analysis linked the genomic structure of prophages to their capacity for carrying virulence genes, while collinearity analysis, combined with 3D structural prediction, identified the broad-spectrum lysozyme Lys81. This lysozyme retains activity under weakly acidic pH and low temperatures, exhibits potent biofilm clearance capability, and demonstrates effective bactericidal activity in organoid models, thereby laying a foundation for the clinical application of MRSA prophages.

Prophages are widely distributed and highly prevalent in *S. aureus*, especially in MRSA lineages. Most clinical MRSA isolates carry multiple prophages, with Sa1, Sa2, and Sa3 as dominant types globally (Goerke et al., 2009). These prophages show strong lineage specificity and are linked to virulence, adaptation, and epidemic success of major MRSA clones (Diep et al., 2006; Verkaik et al., 2011). Consistent with previous reports, intact prophages were detected in 85.7% (Figure 1B) of our 329 MRSA isolates, a prevalence comparable to the 85–95% observed in clinical MRSA isolates (Dini et al., 2019; Ingmer et al., 2019). Furthermore, we found that most MRSA strains across departments and sample sources carried 1–2 intact prophage genomes, with over 60% of strains in each category containing intact prophages (Figures 1C,D). This indicates that intact prophages exhibit a widespread distribution in clinical MRSA isolates, without significant restriction by department or sample source, supporting the applicability of prophages as potential therapeutic targets and providing a theoretical basis for developing therapeutic strategies based on prophages or their products such as endolysins.

To validate the safety of prophage therapy, resistance and virulence gene screening of 515 MRSA prophages revealed a low abundance of resistance genes, with 509 prophages lacking AMR (Figure 2). AMR-negative prophages do not enhance host resistance or transfer VFs, thereby confirming their safety in therapeutic development compared to prophages containing AMR and VFs (Cook and Hynes, 2025; Lehman et al., 2019; Kondo et al., 2021). McCarthy and Lindsay’s studies have demonstrated that horizontal gene transfer (HGT) of virulence genes is regulated by both phage families and host lineages (McCarthy et al., 2012). This assumption was validated multidimensionally in this study: First, the distribution of virulence genes is highly correlated with the genomic structure of prophages (Figures 2B,D), indicating that phylogenetically clustered prophages, sharing similar genomic backgrounds, possess structural characteristics that directly determine their ability to carry VFs (McCarthy et al., 2012; Kondo et al., 2021). Second, correlation analysis of clinical MRSA isolates revealed that the distribution of virulence genes is closely related to the host background, with this uneven distribution explainable by differences in genetic structures such as motifs (Figure 2E). These findings align with the research conclusions of McCarthy and Lindsay, collectively indicating that the acquisition of VFs by prophages is not a random process but is strictly constrained by phage types and bacterial clonal backgrounds. Validation results based on prophages from 118 *S. aureus* strains suggest that, in addition to directly counting virulence genes, the structural characteristics of prophages can serve as a crucial basis for inferring their virulence traits. This insight offers a novel perspective for genetic engineering modifications—prophages with a reduced capacity to carry virulence genes may exhibit enhanced biosafety following intervention.

The study demonstrated that, despite the overall trend in the phylogenetic tree suggesting that prophages are more closely related to temperate phages than to lytic phages and do not cluster with virulent phages (Figure 3A), the phylogenetic analysis of six groups of prophages related to MRSA reference phages revealed that two prophage isolates clustered with their corresponding reference virulent phages. This finding indicates a close evolutionary relationship, which was further corroborated by genetic, collinearity, and homologous protein analyses. Some prophages not only exhibit evolutionary connections with virulent phages but also share conserved functional modules, providing molecular-level evidence for the potential of prophages to infect and lyse MRSA (Figures 3B,C).

This study further identified three conserved homologous proteins—two lysozymes and one phage receptor-binding protein—through collinearity analysis. Two lysozymes (M81_1_37 and M126_1_3) sharing the conserved CHAP and SH3-like domain architecture (Figure 4B) suggest that these functional modules may have arisen from a common ancestral origin or been acquired via horizontal gene transfer. Despite belonging to the same endolysin family, the relatively low overall sequence similarity between M126_1_3 and M81_1_37 implies considerable diversification at the subfamily level, likely driven by genetic drift and variable interdomain linker regions. Notably, phylogenetic analysis positioned M126_1_3 within a basal clade with strong bootstrap support, indicating that M126_1_3 represents an early diverged isoform among the analyzed endolysins (Supplementary Figure 3). Together, these observations highlight the evolutionary plasticity of endolysin structures while supporting M126_1_3 as a distinct, early evolving member with unique phylogenetic characteristics.

Lys81 was priority selected for functional characterization due to its representative CHAP–Amidase_2–SH3_5 domain architecture, which has been associated with broad-spectrum antibacterial activity against diverse pathogens (Kim et al., 2020). Notably, our study identified a strain-dependent bactericidal activity of Lys81 against *S. aureus:* it elicited direct killing in the reference strain ATCC 33591, accompanied by a significant reduction in CFU, whereas in clinical isolates, Lys81 primarily caused severe membrane damage and high mortality without a notable decrease in CFU (Figure 6). The divergent strain-dependent activity of Lys81 may be attributed to strain-specific differences in cell wall structure, membrane composition, and stress response pathways, as widely reported for staphylococcal endolysins and lysozymes (Herbert et al., 2007). Clinical isolates of *S. aureus* frequently exhibit enhanced peptidoglycan modifications, including O-acetylation by OatA, altered wall teichoic acid (WTA) decoration, and increased cross-linking, which block enzymatic hydrolysis by lysins while still permitting membrane perturbation (Bera et al., 2007). Furthermore, variations in cell wall binding domain (CBD)–target interactions and membrane permeability between standard and clinical strains can uncouple membrane damage from CFU-detectable killing (Schmelcher et al., 2012; Becker et al., 2015). Such strain-specific resistance mechanisms are commonly observed in pathogenic staphylococcal and underpin the variable efficacy of phage endolysins against clinical isolates (Sutton et al., 2021). Furthermore, the lytic spectrum of Lys81 confirmed its potent activity against a range of Gram-positive bacteria and considerable efficacy against *P. aeruginosa* strains. Combined with its acid tolerance and optimal activity at pH 5, these characteristics highlight the potential of Lys81 as a promising and adaptable agent for combating infections caused by multiple bacterial pathogens.

Beyond its antibacterial activity, Lys81 exhibited unique thermal and ion regulatory characteristics. Lys81 exhibits a unique wave-like thermal activity curve with three distinct phases: a low-temperature peak at 0°C, a medium-temperature valley (4°C), and a high-temperature rebound (16 and 56–70°C) (Figure 5F). This profile suggests a conformational memory effect, where pre-incubation temperature programs functional state upon cooling (English et al., 2006; Cozzone et al., 1975). The 16°C activity peak aligns with literature-reported conformational transition temperatures (∼12°C) in native lysozyme (Idenyi et al., 2006; Wilson et al., 1997), while reduced activity at 37–42°C indicates a kinetically trapped “pre-denaturation” state. The high-temperature rebound likely reflects transient molten-globule formation with partial refolding during assay (García-Fandiño et al., 2012).

The type and concentration of metal ions exert significant regulatory effects on the lytic activity of Lys81. The observed activation by Na^+^ aligns with previous findings on bacterial and halophilic lysozymes, which commonly exhibit enhanced catalytic activity in the presence of monovalent cations (Madern et al., 2000; Page and Di Cera, 2006; Chang and Carr, 1971). In contrast to Na^+^, in this study, Ca^2 +^ and Mg^2 +^ exhibited biphasic effects on lysozyme activity, with significant activation at 25 mM and marked inhibition above 100 mM (Figure 5G). These concentration thresholds differ from prior reports where optimal activation occurred at 1–10 mM and inhibition was observed at ≥ 1 mM (Elmogy et al., 2015; Boland et al., 2004; Ibrahim et al., 1997), or at high Mg^2 +^ concentrations in T4 lysozyme (Jensen and Kleppe, 1972). Such discrepancies likely reflect variations in enzyme sources (hen egg white versus bacteriophage T4 lysozyme) and assay conditions (pH, substrate type), yet the biphasic modulation pattern remains consistent with classical studies (Ibrahim et al., 1997; Jensen and Kleppe, 1972; Chang and Carr, 1971). Collectively, these findings indicate that Lys81’s ion response not only conforms to general lysozyme regulatory paradigms but also exhibits distinct concentration-dependent specificity, providing experimental evidence for elucidating the ion-mediated regulatory mechanism of this enzyme.

Biofilms serve as a critical protective barrier for bacterial pathogens, shielding them from environmental stressors, host immune responses, and antimicrobial agents. This protective role contributes significantly to the development of recalcitrant persistent infections (Römling and Balsalobre, 2012). Consequently, the capacity to eradicate preformed biofilms has emerged as a pivotal criterion for evaluating novel antibacterial agents. Several endolysins have recently been reported to act on polymicrobial biofilms composed of enterococci and *S. aureus*. For instance, the staphylococcal endolysin LysSA52 achieved a 60% removal of *S. aureus* biofilms following a 12-h incubation at a concentration of 500 μg/mL (Abdurahman et al., 2023). Similarly, Lys84 endolysin eliminated 90% of *S. aureus* biofilms within 2 h at concentrations exceeding 10 μM (Ning et al., 2021). Notably, Ply113 exhibited exceptional biofilm-eradicating potency, achieving up to 95.8% clearance of polymicrobial biofilms at a concentration of 64 μg/mL (Wang et al., 2023). In the present study, Lys81 at 50 μg/mL significantly reduced viable bacteria within MRSA biofilms by 0.69 log_10_ over 5 h, corresponding to an approximate 83% clearance rate (Figure 6C). This 83% viable cell clearance rate indicates effective penetration of the biofilm matrix and the killing of embedded MRSA. Given that biofilms inherently protect bacteria from antimicrobial agents, even modest log_10_ reductions in biofilm-associated CFU hold biological significance. These findings demonstrate that lysozyme exhibits intrinsic antibacterial activity against staphylococcal biofilms, supporting its potential as a candidate agent for managing biofilm-related infections.

*In vitro* monolayer cell cultures have long been employed to evaluate antibacterial efficacy. However, these cultures fail to accurately replicate the complex three-dimensional structure, cellular heterogeneity, and physical barriers present in human respiratory epithelia. Human organoids represent a physiologically relevant model that more closely mimics *in vivo* tissue architecture and host-pathogen interactions (Porotto et al., 2019; Barron et al., 2021; Mahieu et al., 2024; Huang et al., 2026). In this study, we evaluated the antibacterial activity of Lys81 against MRSA using a human pulmonary organoid model. Our findings demonstrated that Lys81 potently reduced bacterial burden in MRSA-infected organoids, while its cytotoxicity displayed distinct organoid-type specificity (Figure 7). Notably, Lys81 displayed unique tissue-specific cytotoxicity and therapeutic profiles that distinguish it from conventional broad-spectrum antibiotics in the human lung organoid system. In normal organoids, mild LDH release was accompanied by an increase in organoid area, consistent with stem cell-mediated epithelial repair mechanisms (Figures 7A,C; Nadkarni et al., 2016). Concomitantly, Lys81 achieved complete bacterial clearance (0 CFU) (Figure 7D). In contrast, Lys81’s antibacterial activity was significantly attenuated in cancer organoids (0.25 log_10_ CFU reduction), a phenotype likely attributed to the hypoxic tumor microenvironment (Figure 7G). This attenuation may result from Lys81’s reliance on intact bacterial membrane structure and function, whereas hypoxia-induced physiological alterations in bacteria could diminish Lys81 sensitivity—paralleling the activity suppression observed with daptomycin under hypoxic conditions (Hess et al., 2013; Miller et al., 2016). Importantly, unlike ciprofloxacin and aminoglycosides, which induce severe epithelial damage (Bredberg et al., 1991; Cojocel et al., 1984; Kaneko et al., 2024), Lys81 demonstrated low toxicity with moderate antibacterial activity in cancer-derived organoids. highlighting its potential as a novel therapeutic strategy for MRSA-infected tumors.

In view of the practical implications of our research, we propose a safety evaluation standard for clinical phage screening by linking prophage genome structure to virulence gene carriage, enabling integration of low-virulence prophage data into databases. A 48-nt conserved motif in high-virulence prophages provides a mechanistic basis (carriage mechanisms unclear). Low-virulence prophage structural traits facilitate therapeutic candidate screening. Homologous protein analysis expanded MRSA phage protein libraries, validating lytic enzyme (Lys81) and recognition protein discovery—this method can extend to co-host phages/prophages to advance phage-bacteria interaction studies.

While our results provide valuable insights into the therapeutic potential of prophage resource libraries for MRSA treatment, several limitations must be addressed. First, the clinical MRSA prophage isolates analyzed in this study were all sourced from a single geographical region, potentially limiting the generalizability of our findings. Second, although we employed a lung organoid model for *in vivo* validation, we have yet to systematically evaluate the long-term efficacy and safety of Lys81 in animal infection models. Furthermore, this study conducted only functional and preliminary characterization of Lys81; M126 has not been functionally validated, and its potential application value requires further clarification. Additionally, we did not assess the risk of Lys81-induced drug resistance. Finally, while the enzyme yield obtained from the current expression system is adequate to support *in vitro* and organoid studies, further optimization is necessary to enhance yield and stability for preclinical or applied research. Based on these limitations, future research should focus on the following directions: (1) Expanding the geographical diversity of samples to verify the universality of prophage distribution; (2) Establishing animal infection models to systematically evaluate the *in vivo* efficacy and long-term biosafety of Lys81; (3) Expressing, purifying, and functionally characterizing M126 to assess its synergistic or complementary potential with Lys81; (4) Conducting serial passage experiments combined with genomic analysis to evaluate the risk and mechanism of drug resistance induction; (5) Improving enzyme yield, stability, and tissue-targeted delivery efficiency through optimization of the expression system and formulation development. These efforts will provide a more comprehensive experimental basis for its eventual clinical application.

## Conclusion

In summary, this study systematically analyzed the epidemiological characteristics of 329 clinical MRSA prophages, revealing the phenomenon of interdepartmental transmission. It also examined their virulence genes and antibiotic resistance genes. Additionally, the safety and efficacy of the prophages as gene engineering vectors were evaluated from a molecular evolutionary perspective. The results indicated a significant correlation between the genomic structure of the prophages and the presence of virulence genes. Collinearity analysis showed that the ancestral prophages and highly virulent MRSA phages exhibit collinear characteristics and share closely related homologous proteins. Through homology functional annotation, two lysozymes and a host-recognition device were identified. Among them, Lys81 (a homolog of N-acetylmuramoyl-L-alanine amidase) was confirmed through prioritized *in vitro* characterization to possess lysozyme activity, demonstrating broad-spectrum antibacterial activity, excellent environmental adaptability, strong biofilm-clearing capability, and good *in vivo* safety and efficacy. This study is the first to validate the safety and efficacy of the identified lysozymes against clinical MRSA isolates in a human lung organ model, providing foundational data and practical references for the clinical translational research of prophage-derived lysozymes against MRSA.

## Data Availability

The datasets presented in this study can be found in online repositories. The names of the repository/repositories and accession number(s) can be found in the article/Supplementary material.

## Footnotes

1
https://meme-suite.org/meme/tools/meme

2
https://blast.ncbi.nlm.nih.gov/Blast.cgi

3
https://www.rcsb.org/search/advanced

4
https://www.uniprot.org/blast/

5
https://www.ncbi.nlm.nih.gov/

6
https://victor.dsmz.de

7
https://itol.embl.de/upload.cgi

8
https://phage.usegalaxy.eu/

9
https://www.ebi.ac.uk/jdispatcher/msa/clustalo

10
https://colab.research.google.com/github/sokrypton/ColabFold/blob/main/AlphaFold2.ipynb

11
http://ekhidna.biocenter.helsinki.fi/dali/

12
https://web.expasy.org/protparam/
