# Supplementary material for: Prophage landscapes in clinical MRSA: safety profiling and discovery of Lys81, a broad-spectrum bacteriolytic enzyme
Source: Front Microbiol. 2026 Aug 31;17:1777609. doi: 10.3389/fmicb.2026.1777609 (PMC13572733; doi:10.3389/fmicb.2026.1777609)
Supplement: Supplementary file 1 [file Data_Sheet_1.zip › Supplementary Material/Supplementary Material.docx]

**1、Nucleotide Sequence of Lys81**

ATGCAAGCAAAATTAACTAAAAAAGAGTTTATAGAGTGGTTGAAAACTTCTGAGGGAAAACAATTCAATGTGGACTTATGGTATGGATTTCAATGCTTTGATTATGCCAATGCTGGTTGGAAAGTTTTGTTTGGATTACTTCTAAAAGGTTTAGGTGCAAAAGATATACCATTTGCAAACAATTTTGATGGACTAGCTACTGTATACCAAAATACGCCGGACTTTTTGGCAAAACCCGGCGATATGGTTGTGTTCGGTAGCAATTACGGTGCAGGATACGGACACGTAGCATGGGTAATTGAAGCAACTTTAGATTATATCATTGTATATGAGCAGAATTGGCTAGGCGGTGGCTGGACTGACAGAATCGAACAACCCGGCTGGGGTTGGGAAAAAGTTACAAGACGACAACATGCTTACGATTTCCCTATGTGGTTTATCCGTCCTAACTTCAAAAGCGAAACAGCTCCACGATCAATACAATCTCCTACGCAAGCATCTAAAAAGGAAACAGCTAAGCCACAACCTAAAGCGGTAGAACTTAAAATTATCAAAGATGTGGTTAAAGGTTATGACCTTCCTAAACGTGGTGGTAATCCTAAGGGTATAGTTATTCATAACGACGCAGGAAGCAAAGGGGCAACAGCAGAAGCGTATCGAAACGGATTAGTTAACGCACCTTTATCAAGATTAGAAGCGGGTATTGCGCATAGTTATGTATCAGGTAACACAGTGTGGCAAGCTTTAGATGAATCGCAAGTAGGTTGGCATACTGCTAACCAATTAGGCAATAAATATTATTACGGTATTGAAGTGTGTCAATCAATGGGAGCGGATAATGCGACGTTTTTAAAAAATGAACAGGCGACTTTCCAAGAATGCGCTAGATTGTTGAAAAAATGGGGATTACCAGCAAACAGAAATACAATCAGATTGCACAATGAATTTACTTCAACATCATGCCCTCATAGAAGTTCGGTTTTACACACTGGTTTTGACCCAGTAACTCGTGGCCTATTGCCAGAAGACAAGCGGTTGCAACTTAAAGACTACTTTATCAAGCAGATTAGGGCGTACATGGATGGTAAAATACCGGTTGCCACTGTCTCTAATGAGTCAAGCGCTTCAAGTAATACAGTTAAACCAGTTGCAAGTGCATGGAAACGTAATAAATATGGTACTTACTACATGGAAGAAAGTGCTAGATTCACAAACGGCAATCAACCAATCACAGTAAGAAAAGTGGGGCCATTCTTATCTTGTCCAGTGGGTTATCAGTTCCAACCTGGTGGGTATTGTGATTATACAGAAGTGATGTTACAAGATGGTCATGTTTGGGTAGGATATACATGGGAGGGGCAACGTTATTACTTGCCTATTAGAACATGGAATGGTTCTGCCCCACCTAATCAGATATTAGGTGACTTATGGGGAGAAATCAGTTAG

**2、Amino acid sequence of Lys81**

MQAKLTKKEFIEWLKTSEGKQFNVDLWYGFQCFDYANAGWKVLFGLLLKGLGAKDIPFANNFDGLATVYQNTPDFLAKPGDMVVFGSNYGAGYGHVAWVIEATLDYIIVYEQNWLGGGWTDRIEQPGWGWEKVTRRQHAYDFPMWFIRPNFKSETAPRSIQSPTQASKKETAKPQPKAVELKIIKDVVKGYDLPKRGGNPKGIVIHNDAGSKGATAEAYRNGLVNAPLSRLEAGIAHSYVSGNTVWQALDESQVGWHTANQLGNKYYYGIEVCQSMGADNATFLKNEQATFQECARLLKKWGLPANRNTIRLHNEFTSTSCPHRSSVLHTGFDPVTRGLLPEDKRLQLKDYFIKQIRAYMDGKIPVATVSNESSASSNTVKPVASAWKRNKYGTYYMEESARFTNGNQPITVRKVGPFLSCPVGYQFQPGGYCDYTEVMLQDGHVWVGYTWEGQRYYLPIRTWNGSAPPNQILGDLWGEIS*

| No. | Strain No. | Bacterial Species |
| --- | --- | --- |
| 1 | ATCC12600 | Type strain of *Staphylococcus aureus* |
| 2 | B80950 | Type strain of S*taphylococcus aureus* |
| 3 | B80951 | Type strain of *Staphylococcus aureus* |
| 4 | B81854 | Type strain of *Staphylococcus aureus* |
| 5 | BMZ137680 | Type strain of *Staphylococcus aureus* |
| 6 | M42 | Methicillin-resistant *Staphylococcus aureus* |
| 7 | M48 | Methicillin-resistant *Staphylococcus aureus* |
| 8 | M56 | Methicillin-resistant *Staphylococcus aureus* |
| 9 | M64 | Methicillin-resistant *Staphylococcus aureus* |
| 10 | M65 | Methicillin-resistant *Staphylococcus aureus* |
| 11 | M67 | Methicillin-resistant *Staphylococcus aureus* |
| 12 | M79 | Methicillin-resistant *Staphylococcus aureus* |
| 13 | M81 | Methicillin-resistant *Staphylococcus aureus* |
| 14 | M96 | Methicillin-resistant *Staphylococcus aureus* |
| 15 | M136 | Methicillin-resistant *Staphylococcus aureus* |
| 16 | M139 | Methicillin-resistant *Staphylococcus aureus* |
| 17 | M170 | Methicillin-resistant *Staphylococcus aureus* |
| 18 | M212 | Methicillin-resistant *Staphylococcus aureus* |
| 19 | M252 | Methicillin-resistant *Staphylococcus aureus* |
| 20 | M257 | Methicillin-resistant *Staphylococcus aureus* |
| 21 | M299 | Methicillin-resistant *Staphylococcus aureus* |
| 22 | M303 | Methicillin-resistant *Staphylococcus aureus* |
| 23 | M304 | Methicillin-resistant *Staphylococcus aureus* |
| 24 | M315 | Methicillin-resistant *Staphylococcus aureus* |
| 25 | M316 | Methicillin-resistant *Staphylococcus aureus* |
| 26 | M321 | Methicillin-resistant *Staphylococcus aureus* |
| 27 | M324 | Methicillin-resistant *Staphylococcus aureus* |
| 28 | M327 | Methicillin-resistant *Staphylococcus aureus* |
| 29 | M329 | Methicillin-resistant *Staphylococcus aureus* |
| 30 | M338 | Methicillin-resistant *Staphylococcus aureus* |
| 31 | M339 | Methicillin-resistant *Staphylococcus aureus* |
| 32 | M346 | Methicillin-resistant *Staphylococcus aureus* |
| 33 | M349 | Methicillin-resistant *Staphylococcus aureus* |
| 34 | M351 | Methicillin-resistant *Staphylococcus aureus* |
| 35 | M352 | Methicillin-resistant *Staphylococcus aureus* |
| 36 | M354 | Methicillin-resistant *Staphylococcus aureus* |
| 37 | M364 | Methicillin-resistant *Staphylococcus aureus* |
| 38 | M366 | Methicillin-resistant *Staphylococcus aureus* |
| 39 | M371 | Methicillin-resistant *Staphylococcus aureus* |
| 40 | M372 | Methicillin-resistant *Staphylococcus aureus* |
| 41 | M373 | Methicillin-resistant *Staphylococcus aureus* |
| 42 | M374 | Methicillin-resistant *Staphylococcus aureus* |
| 43 | M377 | Methicillin-resistant *Staphylococcus aureus* |
| 44 | M378 | Methicillin-resistant *Staphylococcus aureus* |
| 45 | ATCC19606 | Type strain of *Acinetobacter baumannii* |
| 46 | 18-F-58 | *Salmonella Enteritidis* |
| 47 | C106 | *Vibrio vulnificus* |
| 48 | ATCC11775 | Type strain of *Escherichia coli* |
| 49 | B81432 | Type strain of *Listeria monocytogenes* |
| 50 | L2 | *Listeria monocytogenes* |
| 51 | ATCC13883 | Type strain of *Klebsiella pneumoniae* |
| 52 | 19-WJ-644 | *Klebsiella pneumoniae* |
| 53 | ATCC10145 | Type strain of *Pseudomonas aeruginosa* |
| 54 | 20WJ816 | *Pseudomonas aeruginosa* |
| 55 | 2.10E+235 | *Pseudomonas aeruginosa* |
| 56 | 2.10E+238 | *Pseudomonas aeruginosa* |
| 57 | E13 | *Pseudomonas aeruginosa* |
| 58 | E20 | *Pseudomonas aeruginosa* |
| 59 | E24 | *Pseudomonas aeruginosa* |
| 60 | E25 | *Pseudomonas aeruginosa* |
| 61 | P16028 | *Pseudomonas aeruginosa* |
| 62 | P16153 | *Pseudomonas aeruginosa* |

**Supplementary Table 1.** Strains and plasmid used in the experiment

**Supplementary Table 2.** BLAST alignment results of M126_1_3 with homologous sequences

| Query Acc.ver | Subject Acc.ver | % Identity | Alignment Length | E-value | Bit Score | % Positives |
| --- | --- | --- | --- | --- | --- | --- |
| M126_1_3 | WP_070861812.1 | 99.60 | 500 | 0 | 1036 | 100 |
| M126_1_3 | WP_368864113.1 | 99.10 | 442 | 0 | 913 | 99.77 |
| M126_1_3 | WP_408020798.1 | 97.54 | 447 | 0 | 905 | 98.88 |

**Supplementary Table 2.** BLAST alignment results of M126_1_3 with homologous sequences

| Query Acc.ver | Subject Acc.ver | % Identity | Alignment Length | E-value | Bit Score | % Positives |
| --- | --- | --- | --- | --- | --- | --- |
| Lys81 | WP_031784479.1 | 98.69 | 457 | 0 | 945 | 99.34 |
| Lys81 | WP_374746616.1 | 98.16 | 435 | 0 | 892 | 99.08 |
| Lys81 | WP_165811754.1 | 96.67 | 481 | 0 | 972 | 98.13 |
| Lys81 | WP_075344827.1 | 95.41 | 436 | 0 | 868 | 97.71 |
| Lys81 | WP_438293932.1 | 91.87 | 443 | 0 | 858 | 96.16 |
| Lys81 | AWD93103.1 | 88.87 | 485 | 0 | 845 | 90.52 |
| Lys81 | WP_107597703.1 | 82.50 | 480 | 0 | 828 | 89.58 |

**Supplementary Figure 1.** The relationship between the phage sequences with VFs. Every dot in the figure represents a phage sequence, and the color means the count of VFs. The greater the similarity in gene structure, the closer their positions. R² is the fit of the model to the data, and p-value is the correlation between sequence structure and VFs.

**Supplementary Figure 2.** Comparison of predicted protein structures. The Lys81 protein has 3 structural domains: CHAP domain, N-acetylmuramoyl-L-alanine amidase/PGRP domain superfamily, and SH3-like domain. The M126_1_3 protein has 3 structural domains: CHAP domain, N-acetylmuramoyl-L-alanine amidase, catalytic domain, and SH3-like domain.

**Supplementary Figure 3.**The phylogenetic tree of endolysin homologous proteins was constructed using the maximum likelihood method. This tree includes M126_1_3, Lys81, and their homologous sequences identified through BLASTP screening, with a sequence identity of ≥ 80%, totaling 17 protein sequences. Multiple sequence alignment was conducted using MAFFT v7.526 with the parameters set to --auto. The phylogenetic tree was constructed using IQ-TREE v2.0.7, employing the maximum likelihood method, with the optimal substitution model VT+G4 selected automatically based on the Bayesian Information Criterion (BIC). Branch support was assessed through 1000 ultrafast bootstrap tests, displaying only nodes with support ≥ 70%. Tree visualization and formatting were executed using iTOL v6. M126_1_3 forms a sister group with the main lineage containing Lys81, exhibiting 100% support. The internal branches connecting the main lineage demonstrate longer lengths (>2.0 substitutions/position), suggesting an early evolutionary divergence between M126_1_3 and the main lineage, potentially indicating a subtype within the endolysin family.

| query | subject | % identity | alignment length | mismatches | q. start | q. end | s. start | s. end | e-value | bit score |
| --- | --- | --- | --- | --- | --- | --- | --- | --- | --- | --- |
| Lys81 | Lys81 | 100 | 482 | 0 | 1 | 482 | 1 | 482 | 0 | 1003 |
| Lys81 | M126_1_3 | 27.619 | 105 | 62 | 16 | 114 | 37 | 133 | 1.2 | 18.5 |
| Lys81 | M126_1_3 | 54.545 | 11 | 5 | 217 | 227 | 12 | 22 | 3.1 | 16.9 |
| Lys81 | M126_1_3 | 23.858 | 197 | 117 | 85 | 279 | 103 | 268 | 6.5 | 16.2 |
| M126_1_3 | M126_1_3 | 100 | 499 | 0 | 1 | 499 | 1 | 499 | 0 | 1037 |
| M126_1_3 | Lys81 | 54.545 | 11 | 5 | 12 | 22 | 217 | 227 | 3.2 | 16.9 |
| M126_1_3 | Lys81 | 24.771 | 109 | 60 | 37 | 133 | 16 | 114 | 4.3 | 16.5 |
| M126_1_3 | Lys81 | 22.727 | 198 | 118 | 103 | 268 | 85 | 279 | 6.7 | 16.2 |

**Supplementary Table 4**. Table of the bidirectional BLASTP comparison results between Lys81 and M126_1_3.The two full-length protein sequences were subjected to bidirectional alignment using BLASTP version 2.12.0+. Lys81 served as the query sequence for alignment with M126_1_3, while M126_1_3 was used as the query sequence for alignment with Lys81. The scoring matrix utilized was BLOSUM62, with gap penalties set at an open penalty of 11 and an extension penalty of 1. The table provided lists all detected high-scoring alignment fragments (HSPs). The results indicate that no significant full-length coverage matches were identified between Lys81 and M126_1_3, with only a few short fragments exhibiting low scores and high E-values (greater than 1) in localized similarity regions. This finding suggests that no detectable significant homology exists between the two sequences at the full-length level.

**Supplementary Figure 4**. Construction of the recombinant expression plasmid pET-28(a)-Lys81. (A) Agarose gel electrophoresis of the amplifiedLys81gene. M. DL-2000 Marker; Lane 1. Negative control; Lane 2.Lys81gene. (B) Predicted three-dimensional structure of theLys81protein. (C) Agarose gel electrophoresis of the digested empty plasmid pET-28(a). M. DL-5000 Marker; Lanes 1-5. Double-digested pET-28(a); Lane 6. pET-28(a).

| No. | Strain ID | Strain designation | Lytic efficacy |
| --- | --- | --- | --- |
| 1 | ATCC12600 | Type strain of *S.aureus* | + |
| 2 | B80950 | Type strain of *S.aureus* | - |
| 3 | B80951 | Type strain of *S.aureus* | ++ |
| 4 | B81854 | Type strain of *S.aureus* | - |
| 5 | BMZ137680 | Type strain of *S.aureus* | + |
| 6 | M42 | MRSA | + |
| 7 | M48 | MRSA | - |
| 8 | M56 | MRSA | + |
| 9 | M64 | MRSA | + |
| 10 | M65 | MRSA | - |
| 11 | M67 | MRSA | - |
| 12 | M79 | MRSA | - |
| 13 | M81 | MRSA | - |
| 14 | M96 | MRSA | + |
| 15 | M136 | MRSA | - |
| 16 | M139 | MRSA | - |
| 17 | M170 | MRSA | + |
| 18 | M212 | MRSA | + |
| 19 | M252 | MRSA | - |
| 20 | M257 | MRSA | - |
| 21 | M299 | MRSA | - |
| 22 | M303 | MRSA | + |
| 23 | M304 | MRSA | - |
| 24 | M315 | MRSA | - |
| 25 | M316 | MRSA | - |
| 26 | M321 | MRSA | - |
| 27 | M324 | MRSA | - |
| 28 | M327 | MRSA | - |
| 29 | M329 | MRSA | + |
| 30 | M338 | MRSA | - |
| 31 | M339 | MRSA | ++ |
| 32 | M346 | MRSA | + |
| 33 | M349 | MRSA | + |
| 34 | M351 | MRSA | - |
| 35 | M352 | MRSA | + |
| 36 | M354 | MRSA | + |
| 37 | M364 | MRSA | - |
| 38 | M366 | MRSA | + |
| 39 | M371 | MRSA | + |
| 40 | M372 | MRSA | + |
| 41 | M373 | MRSA | + |
| 42 | M374 | MRSA | + |
| 43 | M377 | MRSA | + |
| 44 | M378 | MRSA | + |
| 45 | ATCC19606 | Type strain of *Acinetobacter baumannii* | - |
| 46 | 18-F-58 | *S.Enteritidis* | - |
| 47 | C106 | *V.vulnificus* | + |
| 48 | ATCC11775 | Type strain of *E.coli* | - |
| 49 | B81432 | Type strain of *L.monocytogenes* | + |
| 50 | L2 | *L.monocytogenes* | + |
| 51 | ATCC13883 | Type strain of *K.pneumoniae* | - |
| 52 | 19-WJ-644 | *K.pneumoniae* | - |
| 53 | ATCC10145 | Type strain of *P.aeruginosa* | ++ |
| 54 | 20WJ816 | *P.aeruginosa* | - |
| 55 | 21E234 | *P.aeruginosa* | - |
| 56 | 21E237 | *P.aeruginosa* | - |
| 57 | E13 | *P.aeruginosa* | + |
| 58 | E20 | *P.aeruginosa* | + |
| 59 | E24 | *P.aeruginosa* | - |
| 60 | E25 | *P.aeruginosa* | + |
| 61 | P16028 | *P.aeruginosa* | + |
| 62 | P16153 | *P. aeruginosa* | - |

**Supplementary Table 5.** Determination of the lytic spectrum of Lys81.Note: a lytic activity of 10%-30% is denoted as "+"; a lytic activity of 31%-50% is denoted as "++"; a lytic activity of 51%-70% is denoted as "+++"; a lytic activity of 71%-100% is denoted as "++++"; a lytic activity of less than 10% is denoted as "-."

**Supplementary Figure 5.** In vitro bacteriolytic activity of different concentrations of Lys81 on MRSA 56. Data are presented as mean ± SD (n = 3).

**Supplementary Figure 6.** Temperature-dependent relative activity of lysozyme quantified by ΔOD₆₀₀ assay. The relative activity was calculated based on the change in optical density at 600 nm (ΔOD₆₀₀, defined as the difference between initial and endpoint OD₆₀₀ values of the bacterial suspension after lysozyme treatment). Data represent the mean ± standard deviation (SD) from three independent experiments (each conducted in triplicate), with the maximum ΔOD₆₀₀-derived activity set as 100% relative activity.

**Supplementary Figure 7.** Time-dependent lytic activity of 50 μg/mL Lys81 against the reference strain *S. aureus* ATCC 33591.The OD₆₀₀ of the standard strain culture was monitored over 6 hours. The solid line (ATCC33591) represents the untreated control group, showing stable bacterial growth with no significant decrease in OD₆₀₀ throughout the observation period. The dashed line (Lys81) represents the group treated with 50 μg/mL Lys81, which exhibited a rapid and significant reduction in OD₆₀₀, indicating potent time-dependent lytic activity. Data are presented as mean ± standard deviation (SD).

**Supplementary Figure 8.** Time-dependent lytic activity of 50 μg/mL Lys81 against the Clinical isolate (M172).The OD₆₀₀ of the standard strain culture was monitored over 6 hours. The solid line (M172) represents the untreated control group, showing stable bacterial growth with no significant decrease in OD₆₀₀ throughout the observation period. The dashed line (Lys81) represents the group treated with 50 μg/mL Lys81, which exhibited a rapid and significant reduction in OD₆₀₀, indicating potent time-dependent lytic activity. Data are presented as mean ± standard deviation (SD).
